# Pregnant women’s attitudes and behaviours towards antenatal vaccination against Influenza and COVID-19 in the Liverpool City Region, United Kingdom: Cross-sectional survey

**DOI:** 10.1016/j.jvacx.2023.100387

**Published:** 2023-09-16

**Authors:** Samantha Kilada, Neil French, Elizabeth Perkins, Dan Hungerford

**Affiliations:** a Department of Clinical Infection Microbiology and Immunology, Institute of Infection, Veterinary & Ecological Sciences, University of Liverpool, Liverpool, UK; b Centre for Global Vaccine Research, University of Liverpool, Liverpool, UK; c Institute of Population Health, University of Liverpool, Liverpool, UK

**Keywords:** SARS-CoV-2, COVID-19, Pregnancy, Influenza, Vaccine, Attitudes, Behaviours, Survey, Cross-sectional

## Abstract

**Objectives:**

Influenza poses a serious health risk to pregnant women and their babies. Despite this risk, influenza vaccine uptake in pregnant women in the UK is less than 50%. Little is known about how COVID-19 affects pregnant women, but its management may affect attitudes and behaviours towards vaccination in pregnancy. The study objectives were to establish attitudes and knowledge of pregnant women towards influenza disease and influenza vaccination and to compare these to attitudes and knowledge about COVID-19 and COVID-19 vaccination.

**Design:**

A cross-sectional survey was conducted using an online questionnaire distributed through local advertisement and social media outlets. Information was sought on attitudes and knowledge of influenza and COVID-19 and their respective vaccines.

**Participants and setting:**

Pregnant women residing in Liverpool City Region, UK.

**Results:**

Of the 237 respondents, 73.8% reported receiving an influenza vaccine. Over half (56.5%) perceived themselves to be at risk from influenza, 70.5% believed that if they got influenza, their baby would get ill, and 64.6% believed getting influenza could hurt their baby, 60.3% believed that the influenza vaccine would prevent their baby from getting ill, and 70.8% believed it would protect their baby. Only 32.9% of respondents stated they would receive the COVID-19 vaccine if it were available to them. However, 80.2% stated they would receive a COVID-19 vaccine if they were not pregnant. Most of the women stated that they would accept a vaccine if recommended to them by healthcare professionals.

**Conclusions:**

Acceptance of the influenza and COVID-19 vaccines during pregnancy seems to be more related to the safety of the baby rather than the mother. Women perceived their child to be more at risk than themselves. Information about influenza and COVID-19 vaccine safety as well as healthcare provider recommendations play an important role in vaccine uptake in pregnant women.

## Introduction

Influenza illness is a serious risk to health in pregnant women, especially for those with underlying health conditions. After the H1N1 swine influenza pandemic in 2009, it was recommended that pregnant women in the UK receive the influenza vaccine[1]. However, uptake of the vaccine in pregnant women is consistently below 50% and was 43.5% in the UK for the period between September 2020 and February 2021[2].

Due to immunological and physiological changes associated with pregnancy, pregnant women are at risk of more severe side effects following influenza infection[3]. These adverse side effects include increased risk of miscarriage, premature birth, lowered female growth rates, and increased rate of maternal morbidity and mortality[4], [5], [6].

Inactivated influenza vaccines have an excellent and well-characterised safety profile and can be given at any point during the gestational period with the benefits of vaccine extending to both the high-risk pregnant mother and the infant[7]. Influenza vaccines have been shown to be safe for pregnant women and have no associations with premature birth, low birth weight, or respiratory issues requiring ventilation at birth in infants[8]. Further, evidence suggests transplacental transport of antibodies following maternal vaccination which provide protection for the baby. Giving the influenza vaccine to pregnant women is very effective in preventing lab-confirmed cases of influenza in their infants up to six months of age[9]. A USA-based study found that when women were vaccinated against influenza during pregnancy, there was an 81% decrease in influenza hospitalizations in their babies within the first six months after birth[10].

While there has been some research on influenza and pregnant women, much less is known about the impact of SARS-CoV-2 (severe acute respiratory syndrome coronavirus 2) on pregnant women. In 2020 at the beginning of the COVID-19 (Coronavirus disease 2019) pandemic, there had been limited research at this time regarding the disease caused by the SARS-CoV-2 in pregnant women. Due to immunological and physiological changes in pregnancy, pregnant women may, in theory, be more susceptible to SARS-CoV-2. There may also be an increased risk or compromise to the foetus resulting from low oxygen levels in the mother caused by respiratory illness[11], [12].

A multinational cohort study involving 18 countries (including the UK) found that pregnant women who tested positive for COVID-19 had higher rates of adverse side effects including maternal mortality and preterm births compared to non-infected women[13]. A study from Texas, USA reported that neonatal infection was 3% and these infants were born to asymptomatic or mildly asymptomatic mothers[14]. There is also a possible link between infection with SARS-CoV2 in the third trimester of pregnancy and progressive coagulopathy[15].

On the 16th of April 2021, the Joint Committee on Vaccination and Immunisation (JCVI), UK recommended the use of COVID-19 vaccines for pregnant women in line with the age group specific roll out; prior to this recommendation, the vaccine was not recommended to pregnant women[16]. In a report published in October 2021, almost 20% of critically ill patients with COVID-19 were unvaccinated pregnant women[17]. In response, the NHS (National Health Service) urged all pregnant women to get the COVID-19 vaccine[17]. However, there is a lack of evidence from clinical trials or from reported adverse events from COVID-19 vaccines to fully understand the impact of the COVID-19 vaccine in pregnant women. Given the issues arising from the low uptake of vaccines in pregnancy in the United Kingdom, it is important to understand attitudes towards receiving the vaccine in order to shape future communication strategies targeted at pregnant women. Recent data from the Office of National Statistics reported COVID-19 vaccine hesitancy in women of childbearing age[18]. Concerns about fertility were cited by twenty-five percent of those who reported being unlikely to take the vaccine if offered or who had decided not to take it when offered, 10% of the sample were currently pregnant or trying to get pregnant and 18% were worried about the effect on getting pregnant in the future [18].

In the context of uncertainty about the factors influencing vaccine uptake in pregnant women, we conducted a cross-sectional survey of the attitudes and behaviours of pregnant women in the Liverpool City Region, UK towards influenza illness, COVID-19, and towards antenatal vaccination against influenza and COVID-19. This survey is one part of a larger project regarding vaccine attitudes and behaviours in pregnancy. We explored some of the factors that influence pregnant women’s attitudes towards vaccines and how these attitudes affect vaccine hesitancy or acceptance.

## Methods

### Population and setting

This study only included women who were currently pregnant and living in the Liverpool City Region (LCR) in the North West of England, UK, which includes Liverpool, Knowsley, Sefton, St. Helens, Wirral, and Halton. The LCR contains some of the most deprived areas in England, with over a third of the population living in the 10% most deprived areas of England[19]. In the LCR 2019 census, the majority of the female population ages 16–49 years identified as “White British” (84%) or “White other” (5%)[20]. In 2019, there were 15,045 live births and 15,632 for the year prior[21]. In the LCR, influenza vaccine uptake was 40.5% for September 2020 to February 2021[2].

### Data collection

A questionnaire was developed using JISC (Joint Information Systems Committee) online surveys and was live from 30 October 2020 through 30 April 2021 (Supplementary file 1). This questionnaire was developed using previously created questionnaires as a basis and adding topic-specific questions as they would aid in answering the main question of the study[22]. Additional questions relevant to health behaviours during pregnancy were added. A summary of the study’s purpose, inclusion criteria, confidentiality, and right to withdraw was presented on the first page of the survey prior to obtaining informed consent. The survey included questions examining pregnant women’s health behaviours, such as participation in exercise, the use of antenatal vitamins, and whether or not they smoke. It also included Likert scale questions about the respondent perceptions of illness severity for both influenza and COVID-19 and, were they to contract either, their perceptions of the risks of their own infection on their child as well as their perceptions of the potential risk to others. Questions were also asked about attitudes and beliefs about the influenza vaccine, COVID-19 vaccine, and vaccines in general to understand the factors that lead to vaccine acceptance or hesitancy. As the pertussis vaccination is recommended during pregnancy between 16 and 32 weeks, status of receiving this vaccination was asked[23]. Demographic details were collected on ethnic group, age, occupation, and socioeconomic status. All questions asked in the questionnaire, with the exception of giving consent, were optional and, thus, women could choose not to answer some of the questions. Participants were also asked if they wished to enter an optional prize draw at the end of the questionnaire for the chance to win a £100 Amazon voucher. In order to participate in the draw, they were asked to provide an email address in a separate survey that was not linked to their original responses.

A photo advertisement was created for the study and the link to the questionnaire was provided via multiple social media outlets (Supplementary file 2, Image S1). Social media was used while businesses were closed during the national lockdown in the UK and flyers were used once businesses opened again. Some of these social media outlets included Twitter as well as multiple Facebook groups, including pregnant mother groups in the Liverpool City Region, antenatal class pages, city council pages, charity groups, and community centres. Social media pages were chosen through recommendation by PPI (Patient and Public Involvement) panel and colleagues as well as through search for groups in the area. Flyers were created with a QR code and distributed among local shops, community centres, and places of worship in the area.

### Patient and public involvement

The Institute of Infection, Veterinary, & Ecological Sciences at the University of Liverpool has a patient and public involvement and engagement (PPIE) group which provides an opportunity for discussion about influenza vaccine research. PPIE members had identified reducing inequalities in influenza vaccine uptake as a policy priority. The PPIE panel was used throughout the research process to review study processes and tools. The Liverpool Babies PPIE Group also assisted in the recruitment of the sample by distributing study details and information through their social media and contacts. The findings from this study will be shared through PPIE panels and with maternal and public health services.

### Data analysis

Descriptive analysis was carried out using R version 4.0.3 (R Core Team, Vienna, Austria) and RStudio (Supplementary file 3). We excluded from the analysis any respondents who did not meet the strict eligibility and inclusion criteria; the inclusion criteria were women who are currently pregnant and live in the Liverpool City Region. In the R code, an upper limit of 75 was used to analyse the age question as participants over this age were unlikely to be currently pregnant and therefore did not fit the inclusion criteria.

For the ease of understanding, the Likert scale questions were recoded so that responses of “Strongly Disagree” and “Disagree” were relabelled as “Disagree,” and responses of “Strongly Agree” and “Agree” were relabelled as “Agree.” These questions were analysed and correlated with the demographic questions. Due to the relatively small sample size, questions were collapsed for analysis.

Income was recoded so that responses of “<£10,000,” “£10,001–20,000,” and “£20,001–30,000” were combined and recoded as “≤£30,000,” responses of “£30,001–45,000” and “£45,001–60,000” were recoded as “30,001–60,000,” and the finale category of “>£60,000” remained the same. Occupations were recoded and put into groups of “More advantaged,” “Less advantaged,” or exceptions (full-time students who in the NS-SEC are not classified in the aforementioned groups) based on National Statistics Socio-economic classification (NS-SEC)[24]. The category of “Ethnically Diverse” was recoded to include the following ethnicities: Mixed/multiple ethnic groups (White and Asian, White and Black African, White and Black Caribbean, Other), Asian British/Asian (Chinese, Pakistani, Indian, Bangladeshi, Other), Black British/Black/African/Caribbean, and Other ethnic groups (Arab, Other). Due to the nature of the sampling frame, most statistical analyses were not appropriate but where suitable, differences between continuous variables were assessed using Student’s *t*-test or Wilcoxon rank-sum test if not normally distributed and using chi-squared-test or Fisher’s exact test for categorical variables. Test statistics p-values are presented in the results tables and supplementary tables.

## Results

### Demographics

The total number of survey responses was 252, and, of these, 237 (94%) fulfilled the inclusion criteria. For those excluded, 3/252 (1.2%) were age outliers (elderly or the respondent did not complete their age details) and 12/252 (4.8%) lived outside of the study region. Two-hundred-fourteen of the 237 responses (90.3%) were completed before the recommendation of the COVID-19 vaccine for pregnant women in the UK in April 2021. The age distribution of respondents was from 20 to 43 years old, ($\overset{¯}{x}=$ 31.2 years old; Table 1) and most respondents were over 20 weeks pregnant (median of 27 weeks).

**Table 1:** Demographics for questionnaire respondents in relation to those who were vaccinated/unvaccinated against influenza and those who were accepting, undecided, or against the possibility of the COVID-19 vaccine.

**Demographic Variables**	**Overall** **N = 237 (100%)**	**Vaccinated/ intend to against Influenza** **N = 196 (100%)**	**Unvaccinated against Influenza** **N = 39 (100%)**	**p-value for Influenza Vaccine Status**	**Would have COVID-19 Vaccine** **N = 78 (100%)**	**Undecided about having COVID-19 Vaccine** **N = 38 (100%)**	**Would not have COVID-19 Vaccine** **N = 121 (100%)**	**p-value for willingness to receive COVID-19 Vaccine**
	N (%)	N (%)	N (%)		N (%)	N (%)	N (%)	
**Age**	N = 237	N = 196	N = 39	0.188	N = 78	N = 38	N = 121	0.623
Median [Min, Max]	31 [20, 43]	32 [20, 43]	30 [21, 42]		31.5 [21, 43]	32 [20, 42]	31 [20, 42]	
Q1, Q3	28, 35	28, 35	27, 33.5		27.3, 35	29, 34	38, 34	
**Weeks Pregnant**	N = 235	N = 194	N = 39	<0.001	N = 77	N = 38	N = 120	0.060
Median [Min, Max]	27 [0, 41]	28 [0, 41]	20 [0, 37]		30 [6, 40]	27.5 [0, 39]	24.5 [0, 41]	
Q1, Q3	19, 33	21, 34	15, 29.5		21, 35	19.8, 33	18, 32	
**Occupation**	N = 237	N = 196	N = 39	0.131	N = 78	N = 3	N = 121	0.120
More advantaged groups NS-SEC groups 1–4	189 (79.7)	161 (82.1)	27 (69.2)		64 (82.1)	28 (73.7)	97 (80.2)	
Less advantaged groups NS-SEC groups 5–8	43 (18.1)	32 (16.3)	11 (28.2)		10 (12.8)	10 (26.3)	23 (19)	
Exceptions	5 (2.1)	3 (1.5)	1 (2.6)		4 (5.1)	0 (0)	1 (0.8)	
**Income**	N = 234	N = 193	N = 3	0.351	N = 7	N = 38	N = 11	0.104
≤£30,000	42 (17.9)	31 (16.1)	10 (25.6)		10 (13)	7 (18.4)	25 (21)	
£30,001–60,000	110 (47)	93 (48.2)	16 (41)		31 (40.3)	21 (55.3)	58 (48.7)	
>£60,000	82 (35)	69 (35.7)	13 (33.3)		36 (46.8)	10 (26.3)	36 (30.3)	
**Education**	N = 237	N = 19	N = 39	0.073	N = 78	N = 38	N = 121	NA
GCSE or similar	15 (6.3)	8 (4.1)	7 (17.9)		2 (2.6)	4 (10.5)	9 (7.4)	
NVQ or similar	15 (6.3)	12 (6.1)	3 (7.7)		5 (6.4)	2 (5.3)	8 (6.6)	
A-level or similar	40 (16.9)	34 (17.3)	6 (15.4)		11 (14.1)	9 (23.7)	20 (16.5)	
Undergraduate	66 (27.8)	55 (28.1)	10 (25.6)		24 (30.8)	12 (31.6)	30 (24.8)	
Postgraduate	100 (42.2)	86 (43.9)	13 (33.3)		35 (44.9)	11 (28.9)	54 (44.6)	
Other	1 (0.4)	1 (0.5)	0 (0)		1 (1.3)	0 (0)	0 (0)	
**Ethnicity**	N = 237	N = 196	N = 39	1.000	N = 78	N = 38	N = 121	0.481
White British	215 (90.7)	177 (90.3)	36 (92.3)		73 (93.6)	33 (86.8)	109 (90.1)	
White Other	12 (5.1)	10 (5.1)	2 (5.1)		2 (2.6)	4 (10.5)	6 (5)	
Ethnically Diverse	10 (4.2)	9 (4.6)	1 (2.6)		3 (3.8)	1 (2.6)	6 (5)	
**Number of Children**	N = 237	N = 196	N = 39	0.846	N = 78	N = 38	N = 121	0.469
This is my first	114 (48.1)	93 (47.4)	20 (51.3)		35 (44.9)	19 (50)	60 (49.6)	
1	96 (40.5)	81 (41.3)	14 (35.9)		36 (46.2)	15 (39.5)	45 (37.2)	
2	22 (9.3)	18 (9.2)	4 (10.3)		7 (9)	2 (5.3)	13 (10.7)	
3+	5 (2.1)	4 (2)	1 (2.6)		0 (0)	2 (5.3)	3 (2.5)	
**Number in Household**	N = 235	N = 194	N = 39	0.902	N = 78	N = 38	N = 119	0.709
1	7 (3)	6 (3.1)	1 (2.6)		1 (1.3)	2 (5.3)	4 (3.4)	
2	107 (45.5)	88 (45.4)	18 (46.2)		36 (46.2)	14 (36.8)	57 (47.9)	
3	92 (39.1)	77 (39.7)	14 (35.9)		33 (42.3)	16 (42.1)	43 (36.1)	
4+	29 (12.3)	23 (11.9)	6 (15.4)		8 (10.3)	6 (15.8)	15 (12.6)	
**Smartphone**	N = 235	N = 194	N = 39	NA	N = 77	N = 37	N = 121	NA
Yes	235 (1 0 0)	194 (1 0 0)	39 (1 0 0)		77 (1 0 0)	37 (1 0 0)	121 (1 0 0)	
**Responses by Month**	N = 237	N = 196	N = 39	0.027	N = 78	N = 38	N = 121	NA
November 2020	21 (8.9)	20 (10.2)	1 (2.6)		9 (11.5)	3 (7.9)	9 (7.4)	
December 2020	13 (5.5)	9 (4.6)	4 (10.3)		4 (5.1)	1 (2.6)	8 (6.6)	
January 2021	63 (26.6)	57 (29.1)	5 (12.8)		23 (29.5)	9 (23.7)	31 (25.6)	
February 2021	85 (35.9)	70 (35.7)	14 (35.9)		23 (29.5)	15 (39.5)	47 (38.8)	
March 2021	30 (12.7)	22 (11.2)	8 (20.5)		9 (11.5)	6 (15.8)	15 (12.4)	
April 2021	25 (10.5)	18 (9.2)	7 (17.9)		10 (12.8)	4 (10.5)	11 (9.1)	
**Before/After COVID-19 recommendation**	N = 237	N = 196	N = 39	0.553	N = 78	N = 38	N = 121	0.760
Before	214 (90.3)	178 (90.8)	34 (87.2)		69 (88.5)	34 (89.5)	111 (91.7)	
After	23 (9.7)	18 (9.2)	5 (12.8)		9 (11.5)	4 (10.5)	10 (8.3)	

SD = standard deviation.

NS-SEC = National Statistics Socioeconomic classification.

The majority of the respondents (90.7%) identified themselves as “White British.” Four fifths of respondents (79.7%; n = 189/237) were from more advantaged occupational groups NS-SEC groups 1–4. For household income, 17.9% (n = 42/234) had incomes less than or equal to £30,000 and 47% (n = 110/234) stated their income as between £30,001 and £60,000.

Most of the women who responded to the questionnaire had received the pertussis vaccine (74.7%; n = 177/237), and most also took antenatal vitamins (86.9%; n = 206/237). The majority of women surveyed were non-smokers (96.2%; n = 225/234). The majority of respondents listed themselves as not being in a high-risk group (85.2%; n = 202/237) and 81% (n = 192/237) did not shield during the COVID-19 pandemic. Health behaviours of the included respondents are summarised in Supplementary file 2, Table S1.

### Attitudes and behaviours towards influenza and influenza vaccine

Most women had received the influenza vaccine in their current pregnancy (73.8%; n = 175/237) and 21 (8.9%) had not received the vaccine but intended to do so; 39 women (16.5%) reported that they did not intend to receive the influenza vaccine during their pregnancy (the remaining 2 respondents left this question blank). Of the women who had been vaccinated against influenza or intended to be vaccinated, most (80.6%; n = 158/196) had also received the pertussis vaccine (Supplementary file 2, Table S1). One-hundred-five women (46.1%; n = 105/228) stated receiving the influenza vaccine during a previous pregnancy, and of these women, 14 (13.3%) said they had experienced side effects.

The attitudes and beliefs of these women towards influenza illness as well as their perceived risks of the virus are summarised in Table 2. Less than half of the women (46%; n = 109/237) believed that they would get very ill if they got influenza. For those who were vaccinated or intended to vaccinate, 48.5% (n = 95/196) believed they would get very ill from influenza compared to 33.3% (n = 13/39) of those who were unvaccinated. The majority (70.8%; n = 167/236) believed that if they got influenza, their baby could get ill and 64.6% (n = 153/237) believed it could hurt their baby. However, just over half of the women perceived themselves as being at risk of getting influenza (56.5%; n = 134/237) or that their family/friends were at risk (57.8%; n = 137/237).

**Table 2:** Attitudes and beliefs of pregnant women in the Liverpool City Region, UK towards influenza illness and perceive risks shown in relation to those who are vaccinated/unvaccinated against the virus.

**Questions**	**Overall** **N = 237 (100%)**	**Vaccinated/ intend to against influenza** **N = 196 (100%)**	**Unvaccinated against influenza** **N = 39 (100%)**	**p-value**
	N (%)	N (%)	N (%)	
**If I get the flu, I will get very ill.**	N = 237	N = 196	N = 39	0.026
Disagree	58 (24.5)	41 (20.9)	16 (41)	
Neither Agree or Disagree	70 (29.5)	60 (30.6)	10 (25.6)	
Agree	109 (46)	95 (48.5)	13 (33.3)	
**If I get the flu, I will have to stay home from work/school.**	N = 237	N = 196	N = 39	0.375
Disagree	22 (9.3)	16 (8.2)	6 (15.4)	
Neither Agree or Disagree	28 (11.8)	24 (12.2)	4 (10.3)	
Agree	187 (78.9)	156 (79.6)	29 (74.4)	
**If I get the flu, my baby could get ill.**	N = 236	N = 195	N = 39	0.027
Disagree	25 (10.6)	17 (8.7)	8 (20.5)	
Neither Agree or Disagree	44 (18.6)	34 (17.4)	10 (25.6)	
Agree	167 (70.8)	144 (73.8)	21 (53.8)	
**If I get the flu, it could hurt my baby.**	N = 237	N = 196	N = 39	0.074
Disagree	29 (12.2)	22 (11.2)	7 (17.9)	
Neither Agree or Disagree	55 (23.2)	41 (20.9)	13 (33.3)	
Agree	153 (64.6)	133 (67.9)	19 (48.7)	
**If I get the flu, my other family members or friends could get ill.**	N = 237	N = 196	N = 39	0.197
Disagree	8 (3.4)	6 (3.1)	2 (5.1)	
Neither Agree or Disagree	9 (3.8)	6 (3.1)	3 (7.7)	
Agree	220 (92.8)	184 (93.9)	34 (87.2)	
**If I get the flu, my co-workers/colleagues could get ill.**	N = 237	N = 196	N = 39	0.751
Disagree	12 (5.1)	10 (5.1)	2 (5.1)	
Neither Agree or Disagree	24 (10.1)	19 (9.7)	5 (12.8)	
Agree	201 (84.8)	167 (85.2)	32 (82.1)	
**If I get the flu, I will die.**	N = 237	N = 196	N = 39	0.908
Disagree	196 (82.7)	161 (82.1)	34 (87.2)	
Neither Agree or Disagree	37 (15.6)	31 (15.8)	5 (12.8)	
Agree	4 (1.7)	4 (2)	0 (0)	
**I feel knowledgeable about the flu in general.**	N = 237	N = 196	N = 39	0.223
Disagree	19 (8)	14 (7.1)	5 (12.8)	
Neither Agree or Disagree	52 (21.9)	41 (20.9)	11 (28.2)	
Agree	166 (70)	141 (71.9)	23 (59)	
**I feel knowledgeable about my risk of getting the flu.**	N = 237	N = 196	N = 39	0.301
Disagree	17 (7.2)	12 (6.1)	4 (10.3)	
Neither Agree or Disagree	36 (15.2)	28 (14.3)	8 (20.5)	
Agree	184 (77.6)	156 (79.6)	27 (69.2)	
**I am at risk of getting the flu.**	N = 237	N = 196	N = 39	0.039
Disagree	43 (18.1)	32 (16.3)	11 (28.2)	
Neither Agree or Disagree	60 (25.3)	46 (23.5)	13 (33.3)	
Agree	134 (56.5)	118 (60.2)	15 (38.5)	
**My family and friends are at risk of getting the flu.**	N = 237	N = 196	N = 39	0.139
Disagree	33 (13.9)	24 (12.2)	9 (23.1)	
Neither Agree or Disagree	67 (28.3)	54 (27.6)	12 (30.8)	
Agree	137 (57.8)	118 (60.2)	18 (46.2)	
**You or close friend/family member have had the flu**	N = 237	N = 196	N = 39	0.864
Yes	165 (69.6)	135 (68.9)	28 (71.8)	
No	72 (30.4)	61 (31.1)	11 (28.2)	
**Take over-the-counter medications for the flu or flu-like symptoms**	N = 236	N = 195	N = 39	0.860
Yes	127 (53.8)	106 (54.4)	20 (51.3)	
No	109 (46.2)	89 (45.6)	19 (48.7)	
**Participate in alternative medicine practices for flu treatment or prevention**	N = 236	N = 195	N = 39	0.427
Yes	12 (5.1)	9 (4.6)	3 (7.7)	
No	224 (94.9)	186 (95.4)	36 (92.3)	

Of the vaccinated women, more than half (60.2%; n = 118/196) perceived themselves to be at risk of getting influenza compared to 38.5% (n = 15/39) of the unvaccinated women; of vaccinated women, 67.9% (n = 133/196) believed that if they got influenza, it could hurt their baby compared to 48.7% (n = 19/39) of the unvaccinated women.

The attitudes and beliefs of the pregnant women towards the influenza vaccine are summarised in Table 3. Fifty-three (22.4%) of the 237 women believed they would experience side effects if they receive the influenza vaccine and 99/237 (41.8%) did not believe this. For those who were vaccinated or intended to vaccinate, 33/196 (16.8%) believed they would experience side effects, but a higher proportion of those unvaccinated, 15/39 (38.5%) believed they would experience side effects. The majority of the women (83.5%; n = 198/237) believed that the vaccine would not hurt their baby; for the vaccinated women, 91.8% (n = 180/196) believed it would not hurt their baby compared to 41% (n = 16/39) of the unvaccinated women. In terms of inconvenience or shortages of the vaccine, almost a quarter of the women agreed that it was inconvenient for them to receive the vaccine and 1 in 3 agreed that there was a shortage of the vaccine (24.1%; n = 57/237 and 35%; n = 83/237, respectively). For those vaccinated, 16.3% (n = 31/196) believed it to be inconvenient for them to receive the vaccine compared to 25.6% (n = 10/39) of the unvaccinated women, and 9.7% (n = 19/196) of vaccinated women believed there to be a shortage of the vaccine compared to 10.3% (n = 4/39) of unvaccinated women.

**Table 3:** Attitudes and beliefs of pregnant women in the Liverpool City Region, UK towards the influenza vaccine shown in relation to those who are vaccinated/unvaccinated against the virus.

**Questions**	**Overall** **N = 237 (100%)**	**Vaccinated/ intend to against influenza** **N = 196 (100%)**	**Unvaccinated against influenza** **N = 39 (100%)**	**p-value**
	N (%)	N (%)	N (%)	
**If I have the flu vaccine, I will have side effects from it.**	N = 237	N = 196	N = 39	<0.001
Disagree	99 (41.8)	89 (45.4)	9 (23.1)	
Neither Agree or Disagree	85 (35.9)	74 (37.8)	10 (25.6)	
Agree	53 (22.4)	33 (16.8)	20 (51.3)	
**If I have the flu vaccine, I will get ill from it.**	N = 237	N = 196	N = 39	<0.001
Disagree	177 (74.7)	157 (80.1)	18 (46.2)	
Neither Agree or Disagree	39 (16.5)	33 (16.8)	6 (15.4)	
Agree	21 (8.9)	6 (3.1)	15 (38.5)	
**If I have the flu vaccine, it could hurt my baby.**	N = 237	N = 196	N = 39	<0.001
Disagree	198 (83.5)	180 (91.8)	16 (41)	
Neither Agree or Disagree	34 (14.3)	15 (7.7)	19 (48.7)	
Agree	5 (2.1)	1 (0.5)	4 (10.3)	
**If I have the flu vaccine, it will be painful.**	N = 237	N = 196	N = 39	<0.001
Disagree	177 (74.7)	157 (80.1)	18 (46.2)	
Neither Agree or Disagree	35 (14.8)	24 (12.2)	11 (28.2)	
Agree	25 (10.5)	15 (7.7)	10 (25.6)	
**If I have the flu vaccine, it will not protect me from getting the flu.**	N = 237	N = 196	N = 39	<0.001
Disagree	166 (70)	148 (75.5)	17 (43.6)	
Neither Agree or Disagree	43 (18.1)	33 (16.8)	9 (23.1)	
Agree	28 (11.8)	15 (7.7)	13 (33.3)	
**If I have the flu vaccine, it will not protect my baby.**	N = 236	N = 195	N = 39	<0.001
Disagree	167 (70.8)	154 (79)	11 (28.2)	
Neither Agree or Disagree	54 (22.9)	34 (17.4)	20 (51.3)	
Agree	15 (6.4)	7 (3.6)	8 (20.5)	
**It is inconvenient for me to get the flu vaccine.**	N = 237	N = 196	N = 39	<0.001
Disagree	180 (75.9)	160 (81.6)	18 (46.2)	
Neither Agree or Disagree	15 (6.3)	4 (2)	11 (28.2)	
Agree	42 (17.7)	32 (16.3)	10 (25.6)	
**There is a shortage of the flu vaccine.**	N = 237	N = 196	N = 39	0.368
Disagree	154 (65)	131 (66.8)	22 (56.4)	
Neither Agree or Disagree	60 (25.3)	46 (23.5)	13 (33.3)	
Agree	23 (9.7)	19 (9.7)	4 (10.3)	
**The flu vaccine was recommended to me by my healthcare provider (e.g. doctor, nurse, midwife).**	N = 237	N = 196	N = 39	<0.001
Disagree	19 (8)	9 (4.6)	10 (25.6)	
Neither Agree or Disagree	21 (8.9)	12 (6.1)	8 (20.5)	
Agree	197 (83.1)	175 (89.3)	21 (53.8)	
**If I have the flu vaccine, I will not get ill with the flu.**	N = 237	N = 196	N = 39	0.114
Disagree	87 (36.7)	67 (34.2)	20 (51.3)	
Neither Agree or Disagree	69 (29.1)	59 (30.1)	10 (25.6)	
Agree	81 (34.2)	70 (35.7)	9 (23.1)	
**If I have the flu vaccine, I will help prevent my baby from getting the flu.**	N = 237	N = 196	N = 39	<0.001
Disagree	42 (17.7)	26 (13.3)	15 (38.5)	
Neither Agree or Disagree	52 (21.9)	37 (18.9)	15 (38.5)	
Agree	143 (60.3)	133 (67.9)	9 (23.1)	
**If I have the flu vaccine, I will help prevent my family/friends from getting ill with the flu.**	N = 237	N = 196	N = 39	<0.001
Disagree	48 (20.3)	34 (17.3)	14 (35.9)	
Neither Agree or Disagree	43 (18.1)	31 (15.8)	12 (30.8)	
Agree	146 (61.6)	131 (66.8)	13 (33.3)	
**Received flu vaccine during previous pregnancy**	N = 228	N = 187	N = 39	<0.001
Yes	105 (46.1)	97 (51.9)	7 (17.9)	
No	123 (53.9)	90 (48.1)	32 (82.1)	
**If received flu vaccine in previous pregnancy, were there side effects?**	N = 105	N = 97	N = 7	1.000
Yes	14 (13.3)	13 (13.4)	1 (14.3)	
No	91 (86.7)	84 (86.6)	6 (85.7)	

Questions were posed about perceived effectiveness of the influenza vaccine. Of the respondents, more women believed that the influenza vaccine would prevent family members and friends from getting ill (61.6%; n = 146/237) and their baby from getting ill (60.3%; n = 143/237) than believed it would prevent themselves from getting ill (34.2%; n = 81/237). Just over half of the unvaccinated women (51.3%; n = 20/39) did not believe that the vaccine is effective at preventing them from getting the virus. More vaccinated women (67.9%; n = 133/196) believed that getting the influenza vaccine would help prevent their baby from getting influenza than unvaccinated women (23.1%; n = 9/39). It can also be noted that 51.9% (n = 97/187) of women who reported being vaccinated/intending to vaccinate stated they had received the influenza vaccine during a previous pregnancy compared to 17.9% (n = 7/39) of unvaccinated women.

The means by which the pregnant women were offered the influenza vaccine is shown in Supplementary file 2, Table S2. The vast majority of pregnant women (89.9%; n = 213/237) reported being offered the influenza vaccine and 44.7% (n = 106/237) had it offered by their general practitioner and 46% (n = 109/237) by community services/midwife. More than half (58.2%; n = 138/237) were offered the vaccine in a face-to-face setting.

### Attitudes and behaviours towards vaccines in general

The attitudes and beliefs of the pregnant women towards vaccines in general are shown in Supplementary file 2, Table S3. Most of the respondents across all categories believed vaccines to prevent disease and many more of those who were vaccinated/intended to vaccinate against influenza believed vaccines to be safe (83.7%; n = 164/197) than those who were unvaccinated (56.4%; n = 22/39). A third (33.3%; n = 13/39) of those who were unvaccinated against influenza intended to vaccinate their child against influenza when they are old enough, however most of the women across all categories intended to vaccinate their baby when they are born with all vaccines offered.

The self-reported likelihood of the pregnant women in this study accepting a vaccine varied by the type of healthcare professionals making the recommendation (Supplementary file 2, Table S4). For the healthcare professionals listed, 85.2% (n = 201/236) would accept it from a doctor compared to 68% (n = 157/231) from a pharmacist, 77% (n = 181/235) from a nurse, 84.4% (n = 200/237) from a midwife, and 70.1% (n = 164/234) from a health visitor.

### Attitudes and behaviours towards COVID-19 and COVID-19 vaccine

Of the 237 respondents, 34.2% (n = 81) believed they would get very ill if they got COVID-19 and 20.3% (n = 48) disagreed (Table 4). Almost all of the participants believed they would have to isolate if they became ill with COVID-19 (99.6%; n = 236/237) and all of them believed that if they became ill, their family members and friends with whom they came into contact would have to quarantine. Over three-quarters of the women (78.5%; n = 186/237) believed that if they became ill with COVID-19, their baby could get ill.

**Table 4:** Attitudes and beliefs of pregnant women in the Liverpool City Region, UK towards COVID-19 illness and perceived risks shown in relation to possible COVID-19 vaccine acceptance.

**Questions**	**Overall** **N = 237 (100%)**	**Would have COVID-19 Vaccine** **N = 78 (100%)**	**Undecided about having COVID-19 Vaccine** **N = 38 (100%)**	**Would not have COVID-19 Vaccine** **N = 121 (100%)**	**p-value**
	N (%)	N (%)	N (%)	N (%)	
**If I get COVID-19, I will get very ill.**	N = 237	N = 78	N = 38	N = 121	0.548
Disagree	48 (20.3)	16 (20.5)	5 (13.2)	27 (22.3)	
Neither Agree or Disagree	108 (45.6)	33 (42.3)	22 (57.9)	53 (43.8)	
Agree	81 (34.2)	29 (37.2)	11 (28.9)	41 (33.9)	
**If I get COVID-19, I will have to isolate myself.**	N = 237	N = 78	N = 38	N = 121	1.000
Neither Agree or Disagree	1 (0.4)	0 (0)	0 (0)	1 (0.8)	
Agree	236 (99.6)	78 (1 0 0)	38 (1 0 0)	120 (99.2)	
**If I get COVID-19, my family members and friends who came in contact with me will have to quarantine themselves.**	N = 237	N = 78	N = 38	N = 121	NA
Agree	237 (1 0 0)	78 (1 0 0)	38 (1 0 0)	121 (1 0 0)	
**If I get COVID-19, my baby could get ill.**	N = 237	N = 78	N = 38	N = 121	0.684
Disagree	13 (5.5)	3 (3.8)	1 (2.6)	9 (7.4)	
Neither Agree or Disagree	38 (16)	13 (16.7)	5 (13.2)	20 (16.5)	
Agree	186 (78.5)	62 (79.5)	32 (84.2)	92 (76)	
**If I get COVID-19, my other family members or friends could get ill.**	N = 237	N = 78	N = 38	N = 121	0.666
Disagree	2 (0.8)	0 (0)	0 (0)	2 (1.7)	
Neither Agree or Disagree	7 (3)	2 (2.6)	0 (0)	5 (4.1)	
Agree	228 (96.2)	76 (97.4)	38 (1 0 0)	114 (94.2)	
**I feel knowledgeable about COVID-19 in general.**	N = 237	N = 78	N = 38	N = 121	0.114
Disagree	23 (9.7)	5 (6.4)	1 (2.6)	17 (14)	
Neither Agree or Disagree	37 (15.6)	11 (14.1)	9 (23.7)	17 (14)	
Agree	177 (74.7)	62 (79.5)	28 (73.7)	87 (71.9)	
**I feel knowledgeable about my risk of getting COVID-19.**	N = 237	N = 78	N = 38	N = 121	0.572
Disagree	14 (5.9)	3 (3.8)	1 (2.6)	10 (8.3)	
Neither Agree or Disagree	19 (8)	8 (10.3)	2 (5.3)	9 (7.4)	
Agree	204 (86.1)	67 (85.9)	35 (92.1)	102 (84.3)	
**I am at risk of getting COVID-19.**	N = 237	N = 78	N = 38	N = 121	0.138
Disagree	19 (8)	3 (3.8)	1 (2.6)	15 (12.4)	
Neither Agree or Disagree	38 (16)	15 (19.2)	7 (18.4)	16 (13.2)	
Agree	180 (75.9)	60 (76.9)	30 (78.9)	90 (74.4)	
**My family and friends are at risk of getting COVID-19.**	N = 236	N = 78	N = 38	N = 120	0.256
Disagree	12 (5.1)	2 (2.6)	0 (0)	10 (8.3)	
Neither Agree or Disagree	42 (17.8)	14 (17.9)	8 (21.1)	20 (16.7)	
Agree	182 (77.1)	62 (79.5)	30 (78.9)	90 (75)	
**You or close friend/family member have tested positive for COVID-19**	N = 237	N = 78	N = 38	N = 121	0.491
Yes	149 (62.9)	49 (62.8)	27 (71.1)	73 (60.3)	
No	88 (37.1)	29 (37.2)	11 (28.9)	48 (39.7)	
**You or close friend/family member have been hospitalised for COVID-19**	N = 236	N = 78	N = 38	N = 120	0.534
Yes	29 (12.3)	11 (14.1)	6 (15.8)	12. (10)	
No	207 (87.7)	67 (85.9)	32 (84.2)	108 (90)	

Most of the women stated that they believed themselves to be knowledgeable about COVID-19 and their risks (74.7%; n = 177/237 and 86.1%; n = 204/237, respectively); most also perceived themselves to be at risk of getting ill with the disease (75.9%; n = 180/237). Many of the respondents (62.9%; n = 149/237) had previously tested positive for COVID-19 or had a close friend or family member test positive for COVID-19 (Table 4).

The attitudes and beliefs of the pregnant women participating in this study towards the COVID-19 vaccine are summarised in Table 5. Most of the responses (90.3%; Table 1) to this questionnaire were received before the approval for pregnant women to receive the COVID-19 vaccine on the 16th of April 2021. More than half of respondents stated they were not willing to receive a COVID-19 vaccine if it were available to them (51.1%; n = 121/237). However, the vast majority reported they would be willing to receive the vaccine if they were not pregnant (80.2%; n = 190/237). Of those who would be willing to accept the COVID-19 vaccine, 87.2% (n = 68/78) had received the pertussis vaccine, which is slightly higher than those who had been vaccinated/intended to be vaccinated against influenza (Supplementary file 2, Table S1). Unlike with the influenza vaccine, more women believed that the COVID-19 vaccine would protect themselves than believed it would protect their baby, other family members, or friends (65%; n = 154/237 and 54.9%; n = 130/237, respectively). Eighty-six of the 237 women (36.3%) said they would vaccinate their baby against COVID-19 as soon as possible after they were born, while 35% said (n = 83/237) they would not. Most of the women (68.6%; n = 162/236) said they would get the COVID-19 vaccine every year if it were a seasonal vaccine. When comparing willingness to have the COVID-19 vaccine vs. influenza vaccine status, most responses fell under unwillingness to receive the COVID-19 vaccine or the neither willing nor unwilling regardless of influenza vaccine status (47.4%; n = 93/196 for those who had/intend to have the influenza vaccine and 71.8%; n = 28/39 for those who had not).

**Table 5:** Attitudes and beliefs of pregnant women in the Liverpool City Region, UK towards the COVID-19 vaccine.

**Questions**	**Overall** **N = 237 (100%)**
	N (%)
**If a COVID-19 vaccine was available to me now, I would get it.**	N = 237
Disagree	121 (51.1)
Neither Agree or Disagree	38 (16)
Agree	78 (32.9)
**A COVID-19 vaccine would protect me.**	N = 237
Disagree	28 (11.8)
Neither Agree or Disagree	55 (23.2)
Agree	154 (65)
**A COVID-19 vaccine would protect my baby, other family members, or friends from getting ill with COVID-19.**	N = 237
Disagree	37 (15.6)
Neither Agree or Disagree	70 (29.5)
Agree	130 (54.9)
**I would vaccinate my baby against COVID-19 as soon as possible after they are born.**	N = 237
Disagree	83 (35)
Neither Agree or Disagree	68 (28.7)
Agree	86 (36.3)
**If a COVID-19 vaccine was seasonal, I would get it every year.**	N = 236
Disagree	35 (14.8)
Neither Agree or Disagree	39 (16.5)
Agree	162 (68.6)
**I would get a COVID-19 vaccine if I wasn't pregnant.**	N = 237
Disagree	31 (13.1)
Neither Agree or Disagree	16 (6.8)
Agree	190 (80.2)

The likelihood of the pregnant women accepting the COVID-19 vaccine if recommended to them by different healthcare professionals is shown in Supplementary file 2, Table S5. Most women were willing to accept the COVID-19 vaccine if recommended to them by a doctor (78%; n = 184/236), nurse (64.6%; n = 153/230), or midwife (73.5%; n = 172/234) while less were willing to accept it if recommended by a pharmacist (57.5%; n = 131/228) or health visitor (59.3%; n = 137/231).

## Discussion

The findings of this cross-sectional survey indicate that the majority of respondents had received the influenza vaccine and most believed that the influenza virus would cause more harm to their baby than to themselves. For the attitudes about COVID-19 and the COVID-19 vaccine, only about a third of the women believed they would get very ill from the disease. Unlike with the influenza illness questions, there was less of a divergence in responses to COVID-19 questions. Most of the women felt knowledgeable about COVID-19 and its risks and perceived themselves and their friends and family to be at risk. This is most likely due to the extensive media coverage of COVID-19. Additionally, many of the women stated they would receive the COVID-19 vaccine if they were not pregnant; this may be due to the women not having accessible information regarding the safety of the vaccine during pregnancy. We also found that women who received the pertussis vaccination or received the influenza vaccine during an earlier pregnancy were more likely to be willing to receive the COVID-19 vaccination.

In line with our finding about influenza risk perception, an earlier UK study found that the two main reasons behind influenza vaccine hesitancy in pregnant women were that pregnant women were more likely to implement healthy behaviours (such as not smoking) if they benefitted the baby rather than themselves and that there is a misconception that maternal morbidity and mortality from influenza infection is low[25].

In general, the influenza vaccine uptake for Liverpool City Region is low (less than50%), but for the pregnant women in this study, there was relatively high uptake (82.7% received/intended to receive). Therefore, we have likely accessed a subgroup of pregnant women who are more inclined towards maternal vaccination and more inclined to be willing to participate in research regarding these vaccinations. It is possible that the method of using a cross-sectional survey distributed through the use of social media may have contributed to the access of this group. A study conducted in the USA on COVID-19 vaccine uptake gives examples of how surveys can have biases and overestimate uptake[26]. In this study, it was found that a survey conducted in partnership with Facebook, the Delphi-Facebook COVID-19 Trends and Impact Survey, had the highest level of overestimation compared to the other surveys observed, and it was suggested that some, but not all, of this bias could be due to underrepresentation of less-vaccinated groups[26].

In this group of pregnant women, more than half of them did not believe that they would get very ill from influenza and about a third of the women did not fall into the “Agree” category when asked if getting influenza could hurt their baby. It is important to understand why some women didn’t agree with these statements. It could be related to the information available regarding vaccination or the way that the information about the virus and its risks to them and their baby is provided.

The majority of the women in this study reported feeling knowledgeable about influenza and their risk of getting it. However, only just over half of all the participants felt that they were at risk of getting influenza. Potential reasons for not perceiving themselves at risk could be due to the fact that most of the women in this study reported having received the influenza vaccine or reported intending to receive it, which may lead to them feeling not at risk of the virus as the vaccine is meant to protect them. It could also be due to potential underestimating of the risk of influenza to them as pregnant women. As has been shown, influenza is an underrated health problem in pregnant women, and there is a lack of belief that the vaccine will protect them from influenza infection[27]. A Saudi Arabian study found that poor knowledge about influenza and vaccine safety in pregnant women affected vaccine acceptance, and studies from Australia and Spain stressed the importance of medical professional recommendation on vaccine acceptance during pregnancy[28], [29], [30]. The higher morbidity and mortality effects can be lessened with the aid of education of pregnant women about influenza vaccination[27]. These studies stress the importance of medical staff recommendations and the quality of influenza vaccination information provided to pregnant women on acceptance of the vaccine. Interestingly, most of the women in our study had the vaccine recommended to them by their healthcare provider, which may partly explain why the proportion of women vaccinated was much higher in our study compared to the population average.

As the questionnaire was conducted during the COVID-19 pandemic and a national lockdown in the UK, it can provide insight into the potential effects of the pandemic on attitudes towards influenza vaccination in pregnancy and vaccines in general. Survey distribution occurred mainly before the COVID-19 vaccine was approved for use in pregnant women. Since more of the women reported that they would receive the vaccine if they were not pregnant than those who would receive it during their pregnancy, it is likely that some of the hesitancy towards acceptance of the COVID-19 vaccine during pregnancy is due to the small number of studies in the area at the time of the survey; it also seems that this hesitancy in our study group is more related to the COVID-19 vaccine specifically than to all maternal vaccinations. Indeed, a nationwide cross-sectional survey in Qatar found that 25% of perinatal women were hesitant about receiving a COVID-19 vaccine citing infection risks and safety concerns [31]. A web survey conducted multi-nationally (including the UK) found that of the pregnant and breastfeeding women sampled, hesitancy towards the COVID-19 vaccine was found in 40–50%[32]. More rapid cycle evaluation will need to be conducted on COVID-19 vaccine safety and effectiveness in pregnancy and disseminated efficiently as the safety of the baby is the priority for this group of women. These studies stress the importance of providing timely and accessible information to pregnant women about vaccines during pregnancy. Our study also shows the importance of who provides this information to pregnant women, with doctors and midwives most influential.

### Strengths and limitations

At the time of this study, limited scientific research has been reported on SARS-CoV-2 infection and COVID-19 in pregnant women. Furthermore, clinical trials of the vaccine have yet to be reported in pregnant women. Also, understanding the attitudes of pregnant women toward influenza vaccination, the reasoning for hesitancy, and the importance of medical staff recommendations can assist in developing messages that provide information on the safety and effectiveness of the vaccine. The study identifies the complexity of people’s behaviour in relation to vaccine uptake which is worthy of further investigation.

Our study’s participants reflect the ethnic and socioeconomic structure of the Liverpool City Region[20]. However, the small sample size, the self-reflected nature of the sample, and the high uptake of the influenza vaccine in this sample of pregnant women raise questions about the extent to which the sample is representative of the vaccination behaviours of pregnant women living in the geographical region of Liverpool. Given that influenza vaccine status was self-reported and the anonymous nature of the survey, independent verification of whether this reflected vaccine status as recorded in the health records was not possible. It is, therefore, possible that the reported uptake of the influenza vaccine overestimated the number of women immunised or intending to be immunised. Due to the COVID-19 pandemic, the survey was largely distributed through social media links so we were unable to access women who did not use the social media sites we selected. Future surveys could use NHS sites (with appropriate approvals) to access the pregnant woman population to achieve a more representative population. Finally, due to the small sample size, subgroup analyses of ethnically diverse groups and less advantaged groups were not possible; this small sample size also made more advanced statistical analyses inappropriate.

## Conclusions

Most of the women in this study had received the influenza vaccine during their pregnancy. Concerns raised by mothers about vaccination related more to the safety of their baby rather than themselves. Correct and accessible information on the risks of influenza illness as well as vaccine recommendation, especially from doctors and midwives, plays a huge part in perceptions of vaccine effectiveness and safety in pregnant women. Vaccine hesitancy towards COVID-19 vaccines in this group of pregnant women from Liverpool, UK was associated with not knowing the risks to them or their child of both COVID-19 illness and potential side effects/adverse events from COVID-19 vaccination. This is likely directly related to the paucity of scientific studies in the area. Future surveys should explore the reasons behind vaccine hesitancy and should also focus on less advantaged, hard to reach groups of pregnant women. The next steps in this study will be to conduct focus groups and interviews to gain a deeper understanding of attitudes towards and enablers of influenza and COVID-19 vaccination in pregnancy.

## Declaration of Competing Interest

The authors declare the following financial interests/personal relationships which may be considered as potential competing interests: Daniel Hungerford reports a relationship with GlaxoSmithKline Biologicals Ltd that includes: funding grants. Daniel Hungerford reports a relationship with Seqirus UK Ltd that includes: funding grants. Daniel Hungerford reports a relationship with Merck Sharp & Dohme Corp that includes: funding grants. Neil French reports a relationship with Seqirus UK Ltd that includes: funding grants. Neil French reports a relationship with GlaxoSmithKline Biologicals Ltd that includes: funding grants.

## Data Availability

The authors do not have permission to share data.
