# Supplementary material for: Pregnant women’s attitudes and behaviours towards antenatal vaccination against Influenza and COVID-19 in the Liverpool City Region, United Kingdom: Cross-sectional survey
Source: Vaccine X. 2023 Sep 16;15:100387. doi: 10.1016/j.jvacx.2023.100387 (PMC10518603; doi:10.1016/j.jvacx.2023.100387)
Supplement: Supplementary data 2 [file mmc2.docx]

Supplementary file 2. Supplementary images and tables.

Image S1. Photo advertisement for questionnaire for distribution through social media outlets.


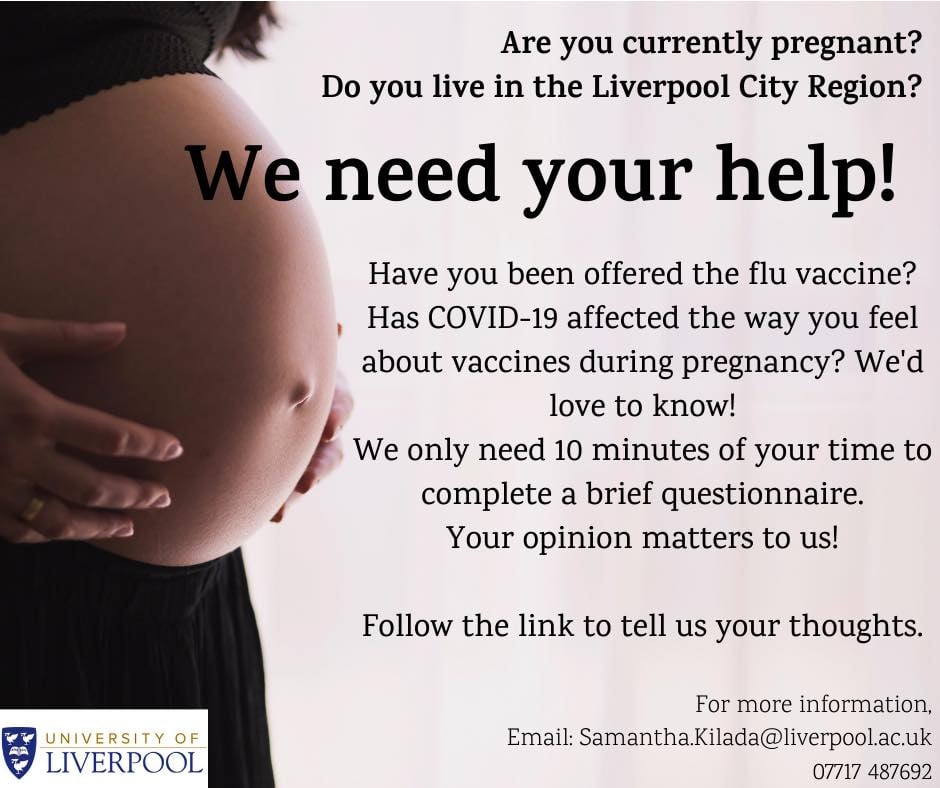


Table S1. Health behaviours for questionnaire respondents in relation to those who were vaccinated/unvaccinated against influenza and those who were accepting, undecided, or against the possibility of the COVID-19 vaccine.

| **Demographic Variables** | **Overall  N=237 (100%)** | **Vaccinated/ intend to against Influenza N=196 (100%)** | **Unvaccinated against Influenza N=39 (100%)** | **p-value for Influenza Vaccine Status** | **Would have COVID-19 Vaccine N=78 (100%)** | **Undecided about having COVID-19 Vaccine N=38 (100%)** | **Would not have COVID-19 Vaccine N=121 (100%)** | **p-value for willingness to receive COVID-19 Vaccine** |
| --- | --- | --- | --- | --- | --- | --- | --- | --- |
|  | N (%) | N (%) | N (%) |  | N (%) | N (%) | N (%) |  |
| **Pertussis Vaccine** | N=237 | N=196 | N=39 | <0.001 | N=78 | N=38 | N=121 | 0.002 |
| Yes | 177 (74.7) | 158 (80.6) | 18 (46.2) |  | 68 (87.2) | 30 (78.9) | 79 (65.3) |  |
| No | 60 (25.3) | 38 (19.4) | 21 (53.8) |  | 10 (12.8) | 8 (21.1) | 42 (34.7) |  |
| **Antenatal Vitamins** | N=237 | N=196 | N=39 | 0.191 | N=78 | N=38 | N=121 | 0.965 |
| Yes | 206 (86.9) | 173 (88.3) | 31 (79.5) |  | 67 (85.9) | 33 (86.8) | 106 (87.6) |  |
| No | 31 (13.1) | 23 (11.7) | 8 (20.5) |  | 11 (14.1) | 5 (13.2) | 15 (12.4) |  |
| **Folic Acid** | N=236 | N=195 | N=39 | 0.940 | N=78 | N=38 | N=120 | 0.875 |
| Yes | 192 (81.4) | 159 (81.5) | 31 (79.5) |  | 63 (80.8) | 30 (78.9) | 99 (82.5) |  |
| No | 44 (18.6) | 36 (18.5) | 8 (20.5) |  | 15 (19.2) | 8 (21.1) | 21 (17.5) |  |
| **High Risk** | N=237 | N=196 | N=39 | 0.221 | N=78 | N=38 | N=121 | 1.000 |
| Yes | 35 (14.8) | 32 (16.3) | 3 (7.7) |  | 12 (15.4) | 5 (13.2) | 18 (14.9) |  |
| No | 202 (85.2) | 164 (83.7) | 36 (92.3) |  | 66 (84.6) | 33 (86.8) | 103 (85.1) |  |
| **Shielded during COVID-19 pandemic** | N=237 | N=196 | N=39 | 1.000 | N=78 | N=38 | N=121 | 0.923 |
| Yes | 45 (19) | 38 (19.4) | 7 (17.9) |  | 15 (19.2) | 8 (21.1) | 22 (18.2) |  |
| No | 192 (81) | 158 (80.6) | 32 (82.1) |  | 63 (80.8) | 30 (78.9) | 99 (81.8) |  |
| **Days per week exercised before pregnancy** | N=236 | N=195 | N=39 | 0.243 | N=77 | N=38 | N=121 | NA |
| 0 | 17 (7.2) | 14 (7.2) | 3 (7.7) |  | 4 (5.2) | 0 (0) | 1 (10.7) |  |
| 1-2 | 97 (41.1) | 83 (42.6) | 13 (33.3) |  | 27 (35.1) | 22 (57.9) | 48 (39.7) |  |
| 3-4 | 77 (32.6) | 58 (29.7) | 18 (46.2) |  | 28 (36.4) | 9 (23.7) | 40 (33.1) |  |
| 5-7 | 45 (19.1) | 40 (20.5) | 5 (12.8) |  | 18 (23.4) | 7 (18.4) | 20 (16.5) |  |
| **Days per week exercise currently** | N=236 | N=195 | N=39 | 0.325 | N=78 | N=38 | N=120 | NA |
| 0 | 56 (23.7) | 49 (25.1) | 7 (17.9) |  | 18 (23.1) | 8 (21.1) | 30 (25) |  |
| 1-2 | 106 (44.9) | 81 (41.5) | 23 (59) |  | 31 (39.7) | 20 (52.6) | 55 (45.8) |  |
| 3-4 | 49 (20.8) | 43 (22.1) | 6 (15.4) |  | 19 (24.4) | 7 (18.4) | 23 (19.2) |  |
| 5-7 | 25 (10.6) | 22 (11.3) | 3 (7.7) |  | 10 (12.8) | 3 (7.9) | 12 (10) |  |
| **Smoker** | N=234 | N=195 | N=37 | 0.038 | N=77 | N=37 | N=120 | 0.170 |
| Yes | 9 (3.8) | 5 (2.6) | 4 (10.8) |  | 1 (1.3) | 3 (8.1) | 5 (4.2) |  |
| No | 225 (96.2) | 190 (97.4) | 33 (89.2) |  | 76 (98.7) | 34 (91.9) | 115 (95.8) |  |
| **Discuss vaccination with partner or family member** | N=236 | N=195 | N=39 | 0.045 | N=78 | N=38 | N=120 | 0.426 |
| Yes | 132 (55.9) | 103 (52.8) | 28 (71.8) |  | 41 (52.6) | 19 (50) | 72 (60) |  |
| No | 104 (44.0) | 92 (47.2) | 11 (28.2) |  | 37 (47.4) | 19 (50) | 48 (40) |  |

Table S2. Means by which pregnant women in the Liverpool City Region, UK were offered the influenza vaccine.

| **Questions** | **Overall N=237** |
| --- | --- |
|  | N (%) |
| **Offered flu vaccine** |  |
| Yes | 213 (89.9) |
| No | 24 (10.1) |
| **Offered flu vaccine by GP** |  |
| Yes | 106 (44.7) |
| **Offered flu vaccine by Women's Hospital** |  |
| Yes | 15 (6.3) |
| **Offered flu vaccine by community services/midwife** |  |
| Yes | 109 (46.0) |
| **Offered flu vaccine by health visitor** |  |
| Yes | 7 (3) |
| **Offered flu vaccine by pharmacist** |  |
| Yes | 7 (3) |
| **Offered flu vaccine by "other"** |  |
| Yes | 39 (16.5) |
| **Offered flu vaccine by "other" specify** |  |
| Employer | 36 (15.2) |
| Had to request it | 1 (0.4) |
| NHS maternity email updates | 1 (0.4) |
| Whiston Hospital | 1 (0.4) |
| **Offered by letter** |  |
| Yes | 33 (13.9) |
| **Offered by text** |  |
| Yes | 40 (16.9) |
| **Offered face to face** |  |
| Yes | 138 (58.2) |
| **Offered by other means** |  |
| Yes | 46 (19.4) |
| **Other means specify** |  |
| A leaflet | 1 (0.4) |
| E-mail | 16 (6.8) |
| Booked/requested themselves | 4 (1.7) |
| In work on text | 1 (0.4) |
| Intranet from employer | 1 (0.4) |
| Midwife suggestion | 2 (0.8) |
| Online booking system through trust intranet | 1 (0.4) |
| Telephone | 18 (7.6) |
| Work | 2 (0.8) |

GP = general practitioner

Table S3. Attitudes and beliefs of pregnant women in the Liverpool City Region, UK towards vaccines in general shown in relation to those who were vaccinated/unvaccinated against influenza and those who were accepting, undecided, or against the possibility of the COVID-19 vaccine.

| **Questions** | **Overall  N=237 (100%)** | **Vaccinated/ intend to against Influenza N=196 (100%)** | **Unvaccinated against Influenza N=39 (100%)** | **p-value for Influenza Vaccine Status** | **Would have COVID-19 Vaccine N=78 (100%)** | **Undecided about having COVID-19 Vaccine N=38 (100%)** | **Would not have COVID-19 Vaccine N=121 (100%)** | **p-value for willingness to receive COVID-19 Vaccine** |
| --- | --- | --- | --- | --- | --- | --- | --- | --- |
|  | N (%) | N (%) | N (%) |  | N (%) | N (%) | N (%) |  |
| **Vaccines prevent disease.** | N=237 | N=196 | N=39 | 0.004 | N=78 | N=38 | N=121 | 0.433 |
| Disagree | 21 (8.9) | 16 (8.2) | 5 (12.8) |  | 5 (6.4) | 3 (7.9) | 13 (10.7) |  |
| Neither Agree or Disagree | 23 (9.7) | 14 (7.1) | 9 (23.1) |  | 5 (6.4) | 6 (15.8) | 12 (9.9) |  |
| Agree | 193 (81.4) | 166 (84.7) | 25 (64.1) |  | 68 (87.2) | 29 (76.3) | 96 (79.3) |  |
| **Vaccines are safe.** | N=237 | N=196 | N=39 | <0.001 | N=78 | N=38 | N=121 | <0.001 |
| Disagree | 3 (1.3) | 1 (0.5) | 2 (5.1) |  | 0 (0) | 1 (2.6) | 2 (1.7) |  |
| Neither Agree or Disagree | 46 (19.4) | 31 (15.8) | 15 (38.5) |  | 3 (3.8) | 6 (15.8) | 37 (30.6) |  |
| Agree | 188 (79.3) | 164 (83.7) | 22 (56.4) |  | 75 (96.2) | 31 (81.6) | 82 (67.8) |  |
| **I intend to vaccinate my child with the flu vaccine when they are old enough.** | N=237 | N=196 | N=39 | <0.001 | N=78 | N=38 | N=121 | <0.001 |
| Disagree | 23 (9.7) | 9 (4.6) | 14 (35.9) |  | 1 (1.3) | 0 (0) | 22 (18.2) |  |
| Neither Agree or Disagree | 26 (11) | 14 (7.1) | 12 (30.8) |  | 3 (3.8) | 5 (13.2) | 18 (14.9) |  |
| Agree | 188 (79.3) | 173 (88.3) | 13 (33.3) |  | 74 (94.9) | 33 (86.8) | 81 (66.9) |  |
| **I intend to vaccinate my baby when they are born with all vaccines offered.** | N=237 | N=196 | N=39 | <0.001 | N=78 | N=38 | N=121 | 0.287 |
| Disagree | 3 (1.3) | 0 (0) | 3 (7.7) |  | 0 (0) | 1 (2.6) | 2 (1.7) |  |
| Neither Agree or Disagree | 7 (3) | 2 (1) | 5 (12.8) |  | 1 (1.3) | 0 (0) | 6 (5) |  |
| Agree | 227 (95.8) | 194 (99) | 31 (79.5) |  | 77 (98.7) | 37 (97.4) | 113 (93.4) |  |
| **I am more likely to have a vaccine if my family members or friends have had it.** | N=236 | N=196 | N=38 | 0.133 | N=78 | N=37 | N=121 | 0.171 |
| Disagree | 78 (33.1) | 60 (30.6) | 18 (47.4) |  | 21 (26.9) | 13 (35.1) | 44 (36.4) |  |
| Neither Agree or Disagree | 77 (32.6) | 66 (33.7) | 10 (26.3) |  | 33 (42.3) | 8 (21.6) | 36 (29.8) |  |
| Agree | 81 (34.3) | 70 (35.7) | 10 (26.3) |  | 24 (30.8) | 16 (43.2) | 41 (33.9) |  |

Table S4. The likelihood of pregnant women in the Liverpool City Region, UK to accept a vaccine if recommended by different healthcare professionals.

| **I am more likely to have a vaccine if it is recommended by a:** | **Overall N=237 (100%)** | **Vaccinated/ intend to against influenza N=196 (100%)** | **Unvaccinated against influenza N=39 (100%)** | **p-value** |
| --- | --- | --- | --- | --- |
|  | N (%) | N (%) | N (%) |  |
| **Doctor** | N=236 | N=195 | N=39 | <0.001 |
| Disagree | 6 (2.5) | 3 (1.5) | 3 (7.7) |  |
| Neither Agree or Disagree | 29 (12.3) | 15 (7.7) | 14 (35.9) |  |
| Agree | 201 (85.2) | 177 (90.8) | 22 (56.4) |  |
| **Pharmacist** | N=231 | N=191 | N=38 | <0.001 |
| Disagree | 21 (9.1) | 15 (7.9) | 6 (15.8) |  |
| Neither Agree or Disagree | 53 (22.9) | 33 (17.3) | 20 (52.6) |  |
| Agree | 157 (68) | 143 (74.9) | 12 (31.6) |  |
| **Nurse** | N=235 | N=194 | N=39 | <0.001 |
| Disagree | 10 (4.3) | 3 (1.5) | 7 (17.9) |  |
| Neither Agree or Disagree | 44 (18.7) | 27 (13.9) | 17 (43.6) |  |
| Agree | 181 (77) | 164 (84.5) | 15 (38.5) |  |
| **Midwife** | N=237 | N=196 | N=39 | <0.001 |
| Disagree | 8 (3.4) | 2 (1) | 6 (15.4) |  |
| Neither Agree or Disagree | 29 (12.2) | 15 (7.7) | 14 (35.9) |  |
| Agree | 200 (84.4) | 179 (91.3) | 19 (48.7) |  |
| **Health visitor** | N=234 | N=193 | N=39 | <0.001 |
| Disagree | 22 (9.4) | 12 (6.2) | 10 (25.6) |  |
| Neither Agree or Disagree | 48 (20.5) | 29 (15) | 19 (48.7) |  |
| Agree | 164 (70.1) | 152 (78.8) | 10 (25.6) |  |
| **Family member or friend** | N=231 | N=190 | N=39 | 0.032 |
| Disagree | 57 (24.7) | 44 (23.2) | 13 (33.3) |  |
| Neither Agree or Disagree | 90 (39) | 70 (36.8) | 19 (48.7) |  |
| Agree | 84 (36.4) | 76 (40) | 7 (17.9) |  |

Table S5. The likelihood of pregnant women in the Liverpool City Region, UK to accept the COVID-19 vaccine if recommended by different healthcare professionals.

| **I am more likely to have a COVID-19 vaccine if it is recommended to me by a:** | **Overall N=237 (100%)** | **Would have COVID-19 Vaccine N=78 (100%)** | **Undecided about having COVID-19 Vaccine N=38 (100%)** | **Would not have COVID-19 Vaccine N=121 (100%)** | **p-value** |
| --- | --- | --- | --- | --- | --- |
|  | N (%) | N (%) | N (%) | N (%) |  |
| **Doctor** | N=236 | N=78 | N=38 | N=120 | <0.001 |
| Disagree | 21 (8.9) | 0 (0) | 0 (0) | 21 (17.5) |  |
| Neither Agree or Disagree | 31 (13.1) | 8 (10.3) | 5 (13.2) | 18 (15.0) |  |
| Agree | 184 (78) | 70 (89.7) | 33 (86.8) | 81 (67.5) |  |
| **Pharmacist** | N=228 | N=76 | N=37 | N=115 | <0.001 |
| Disagree | 40 (17.5) | 1 (1.3) | 1 (2.7) | 38 (33) |  |
| Neither Agree or Disagree | 57 (25) | 12 (15.8) | 10 (27) | 35 (30.4) |  |
| Agree | 131 (57.5) | 63 (82.9) | 26 (70.3) | 42 (36.5) |  |
| **Nurse** | N=230 | N=76 | N=36 | N=110 | <0.001 |
| Disagree | 34 (14.3) | 0 (0) | 1 (2.6) | 33 (27.3) |  |
| Neither Agree or Disagree | 43 (18.1) | 10 (12.8) | 9 (23.7) | 24 (19.8) |  |
| Agree | 153 (64.6) | 66 (84.6) | 26 (68.4) | 61 (50.4) |  |
| **Midwife** | N=234 | N=76 | N=38 | N=120 | <0.001 |
| Disagree | 27 (11.5) | 0 (0) | 0 (0) | 27 (22.5) |  |
| Neither Agree or Disagree | 35 (15) | 9 (11.8) | 6 (15.8) | 20 (16.7) |  |
| Agree | 172 (73.5) | 67 (88.1) | 32 (84.2) | 73 (60.8) |  |
| **Health visitor** | N=231 | N=74 | N=38 | N=119 | <0.001 |
| Disagree | 42 (18.2) | 2 (2.7) | 4 (10.5) | 36 (30.3) |  |
| Neither Agree or Disagree | 52 (22.5) | 11 (14.9) | 10 (26.3) | 31 (25.8) |  |
| Agree | 137 (59.3) | 61 (82.4) | 24 (63.2) | 52 (43.7) |  |
| **Family member or friend** | N=227 | N=74 | N=38 | N=115 | <0.001 |
| Disagree | 74 (32.6) | 9 (12.2) | 13 (34.2) | 52 (45.2) |  |
| Neither Agree or Disagree | 84 (37) | 32 (43.2) | 17 (44.7) | 35 (30.4) |  |
| Agree | 69 (30.4) | 33 (44.6) | 8 (21.1) | 28 (24.3) |  |
