# Supplementary material for: Pregnant women’s attitudes and behaviours towards antenatal vaccination against Influenza and COVID-19 in the Liverpool City Region, United Kingdom: Cross-sectional survey
Source: Vaccine X. 2023 Sep 16;15:100387. doi: 10.1016/j.jvacx.2023.100387 (PMC10518603; doi:10.1016/j.jvacx.2023.100387)
Supplement: Supplementary data 3 [file mmc3.docx]

Supplementary File 3. R code.

a <- file.choose()

setwd(dirname(a))

require(ggplot2)

require(xlsx)

require(stringr)

require(data.table)

require(ISOweek)

require(ggplot2)

require(epitools)

require(scales)

require(surveillance)

require(Hmisc)

require(dplyr)

require(readxl)

require(stringr)

library(ggpubr)

require(readxl)

require(finalfit)

require(table1)

require(arsenal)

library(extrafont)

cbPalette <- c("blue","red", "pink", "brown", "black", "green","grey", "orange","yellow","dark red") ##PHE mid color palette

theme_set(theme_bw(base_size = 12, base_family="times"))

coded <- read_excel("questionnaire 1 coded responses.xlsx", sheet = 1, trim_ws = T, col_types = NULL)

coded2<-coded[c("URN","Q1", "Q2", "Q3", "Q4", "Q5", "Q6", "Q6_a", "Q7", "Q8", "Q9", "Q10", "Q11_1", "Q11_2", "Q11_3", "Q11_4", "Q11_a", "Q12", "Q13_1", "Q13_2", "Q13_3", "Q13_4", "Q13_5", "Q13_6", "Q14", "Q15", "Q16", "Q17", "Q18", "Q19", "Q22", "Q22_a_1", "Q22_a_2", "Q22_a_3", "Q22_a_4", "Q22_a_5", "Q22_a_6", "Q22_a_i", "Q22_b_1", "Q22_b_2", "Q22_b_3", "Q22_b_4", "Q22_b_i", "Q23", "Q23_a", "Q24", "Q24_a", "Q25", "Q26", "Q27", "Q28", "Q29_1", "Q29_2", "Q34", "Q35", "Q36", "Q41_1", "Q41_2", "Q41_3", "Q41_4", "Q41_5", "Q41_6", "Q41_7", "Q41_8", "Q41_a", "CompletionDate")]

noncoded <- read.csv("questionnaire 1 non-coded responses.csv", fileEncoding = "latin1")

noncoded2 <- noncoded[c("Unique.Response.Number","X20.1..If.I.get.the.flu..I.will.get.very.ill.", "X20.2..If.I.get.the.flu..I.will.have.to.stay.home.from.work.school.", "X20.3..If.I.get.the.flu..my.baby.could.get.ill.", "X20.4..If.I.get.the.flu..it.could.hurt.my.baby.", "X20.5..If.I.get.the.flu..my.other.family.members.or.friends.could.get.ill.", "X20.6..If.I.get.the.flu..my.co.workers.colleagues.could.get.ill.", "X20.7..If.I.get.the.flu..I.will.die.", "X21.1..I.feel.knowledgeable.about.the.flu.in.general.", "X21.2..I.feel.knowledgeable.about.my.risk.of.getting.the.flu.", "X21.3..I.am.at.risk.of.getting.the.flu.", "X21.4..My.family.and.friends.are.at.risk.of.getting.the.flu.", "X30.1..If.I.have.the.flu.vaccine..I.will.have.side.effects.from.it.", "X30.2..If.I.have.the.flu.vaccine..I.will.get.ill.from.it.", "X30.3..If.I.have.the.flu.vaccine..it.could.hurt.my.baby.", "X30.4..If.I.have.the.flu.vaccine..it.will.be.painful.", "X30.5..If.I.have.the.flu.vaccine..it.will.not.protect.me.from.getting.the.flu.", "X30.6..If.I.have.the.flu.vaccine..it.will.not.protect.my.baby.", "X30.a.1..It.is.inconvenient.for.me.to.get.the.flu.vaccine.", "X30.a.2..There.is.a.shortage.of.the.flu.vaccine.", "X30.a.3..The.flu.vaccine.was.recommended.to.me.by.my.healthcare.provider..e.g..doctor..nurse..midwife..", "X31.1..If.I.have.the.flu.vaccine..I.will.not.get.ill.with.the.flu.", "X31.2..If.I.have.the.flu.vaccine..I.will.help.prevent.my.baby.from.getting.the.flu.", "X31.3..If.I.have.the.flu.vaccine..I.will.help.prevent.my.family.friends.from.getting.ill.with.the.flu.", "X32.1..Vaccines.prevent.disease.", "X32.2..Vaccines.are.safe.", "X32.3..I.intend.to.vaccinate.my.child.with.the.flu.vaccine.when.they.are.old.enough.", "X32.4..I.intend.to.vaccinate.my.baby.when.they.are.born.with.all.vaccines.offered.", "X32.5..I.am.more.likely.to.have.a.vaccine.if.my.family.members.or.friends.have.had.it.", "X33.1....doctor", "X33.2....pharmacist", "X33.3....nurse", "X33.4....midwife", "X33.5....health.visitor", "X33.6....family.member.or.friend", "X37.1..If.I.get.COVID.19..I.will.get.very.ill.", "X37.2..If.I.get.COVID.19..I.will.have.to.isolate.myself.", "X37.3..If.I.get.COVID.19..my.family.members.and.friends.who.came.in.contact.with.me.will.have.to.quarantine.themselves.", "X37.4..If.I.get.COVID.19..my.baby.could.get.ill.", "X37.5..If.I.get.COVID.19..my.other.family.members.or.friends.could.get.ill.", "X38.1..I.feel.knowledgeable.about.COVID.19.in.general.", "X38.2..I.feel.knowledgeable.about.my.risk.of.getting.COVID.19.", "X38.3..I.am.at.risk.of.getting.COVID.19.", "X38.4..My.family.and.friends.are.at.risk.of.getting.COVID.19", "X39.1..If.a.COVID.19.vaccine.was.available.to.me.now..I.would.get.it.", "X39.2..A.COVID.19.vaccine.would.protect.me.", "X39.3..A.COVID.19.vaccine.would.protect.my.baby..other.family.members..or.friends.from.getting.ill.with.COVID.19.", "X39.4..I.would.vaccinate.my.baby.against.COVID.19.as.soon.as.possible.after.they.are.born.", "X39.5..If.a.COVID.19.vaccine.was.seasonal..I.would.get.it.every.year.", "X39.6..I.would.get.a.COVID.19.vaccine.if.I.wasn.t.pregnant.", "X40.1....doctor.", "X40.2....pharmacist.", "X40.3....nurse.", "X40.4....midwife.", "X40.5....health.visitor.", "X40.6....family.member.or.friend")]

noncoded[noncoded==""]<-NA

full<-cbind(coded2,noncoded2)

#rename columns

colnames(full)

names <- c("URN", "Consent", "Age", "Weeks.pregnant", "No.of.children", "High.risk", "Education.Lvl", "Education.other", "Occupation", "Postcode", "Income", "Smartphone", "Access.GP.online.appts", "Access.GP.online.prescrp", "Access.GP.online.records", "Access.GP.online.other", "Access.other.specify", "No.in.household", "Live.with.alone", "Live.with.partner", "Live.with.child.ren", "Live.with.parent.s", "Live.with.other.family", "Live.with.roommate.s", "Ethnicity", "Smoker", "Exercise.before", "Exercise.currently", "Antenatal.vitamins", "Folic.acid", "Offered.flu.vacc", "If.yes.offered.GP", "If.yes.offered.wo.hosp", "If.yes.offered.comm.ser.midwife", "If.yes.offered.health.vis", "If.yes.offered.pharm", "If.yes.offered.other", "Who.other.offered.specify", "How.offered.letter", "How.offered.text", "How.offered.face.to.face", "How.offered.other", "How.other.offered.specify", "Flu.vacc.this.preg", "If.no.intend", "Flu.vacc.prev.preg", "If.yes.side.effects", "Discuss.vacc.with.partner.fam", "You.or.close.friend.fam.flu", "OTC.meds.flu", "Alt.med.flu", "Pertuss.vacc.yes", "Pertuss.vacc.no", "You.or.close.friend.fam.pos.COVID", "You.or.close.friend.fam.hosp.COVID", "Shield", "Heard.Uni.site", "Heard.GP.prac", "Heard.Mumsnet", "Heard.Netmums", "Heard.Bambis", "Heard.Liv.Mums", "Heard.fam.friend", "Heard.other", "Heard.ques.Other.specify", "Completion.Date","Unique.Response.Number", "Flu.ill", "Flu.stay.home.work.school", "Flu.baby.ill", "Flu.hurt.baby", "Flu.family.friends.ill", "Flu.coworkers.colleages.ill", "Flu.die", "Knowledgeable.flu.general", "Knowledgeable.flu.risk", "I.am.risk.getting.flu", "Family.friends.risk.getting.flu", "Flu.vacc.side.effects", "Flu.vacc.ill", "Flu.vacc.hurt.baby", "Flu.vacc.painful", "Flu.vacc.not.protect.me", "Flu.vacc.not.protect.baby", "Inconvenient.flu.vacc", "Shortage.flu.vacc", "Flu.vacc.recommended", "Flu.vacc.not.get.ill", "Flu.vacc.prevent.baby.ill", "Flu.vacc.prevent.family.friends.ill", "Vacc.prevent.disease", "Vacc.safe", "Intend.vacc.child.flu", "Intend.vacc.baby", "More.likely.vacc.if.family.friends.have", "Doc.flu", "Pharm.flu", "Nurse.flu", "Midwife.flu", "Health.visitor.flu", "Fam.friend.flu", "COVID.ill", "COVID.isolate", "COVID.friends", "COVID.could.contact.quarantine", "COVID.family.friends.contact.quarantine", "Knowledge.COVID.general", "Knowledge.COVID.risk", "I.am.risk.getting.COVID", "Family.friends.risk.getting.COVID", "If.COVID.vacc.available.would.have", "COVID.vacc.protect.me", "COVID.vacc.protect.baby.fam.friends", "Would.vacc.baby.COVID", "If.COVID.vacc.seasonal.would.have", "Would.have.COVID.vacc.if.not.preg", "Doc.COVID", "Pharm.COVID", "Nurse.COVID", "Midwife.COVID", "Health.visitor.COVID", "Fam.friend.COVID")

colnames(full) <- names

#set date fields

full$Completion.Date <- as.Date(full$Completion.Date, origin = "1899-12-30")

full$yearmonth<-format(full$Completion.Date,"%Y%m")

#Pre post April 16th

full$PostApril16<-0

full$PostApril16[full$Completion.Date>="2021-04-16"]<-1

#completion date

full$monthresponse<-format(full$Completion.Date, '%B')

full$monthresponse<-as.factor(full$monthresponse)

#check number of rows

nrow(full)

#remove outliers for age

full$Age <- as.numeric(full$Age)

full1 <- subset(full, Age<70&!is.na(Age))

#postcodes

full1$Postcode<-substr(full1$Postcode, 1, 4)

full1$Postcode<-toupper(full1$Postcode)

full1 <- subset(full1, subset=(Postcode!="CH7" & Postcode!="WN6" & Postcode!="CH66" & Postcode!="WA1" & Postcode!="CW7" & Postcode!="CW1" & Postcode!="PR5" & Postcode!="GU34" & Postcode!="HU6"))

#relabel income

full1$Income<-as.factor(full1$Income)

levels(full1$Income)<-c("<£10,000", "£10,001-20,000", "£20,001-30,000","£30,001-45,000","£45,001-60,000",">£60,000")

##create a new field for income and recode

full1$Income2<-as.character(full1$Income)

full1$Income2[full1$Income2 =="<£10,000" | full1$Income2 =="£10,001-20,000" | full1$Income2 =="£20,001-30,000"]<-"<=£30,000"

full1$Income2[full1$Income =="£30,001-45,000" |full1$Income =="£45,001-60,000"]<-"£30,001-60,000"

full1$Income2<-as.factor(full1$Income2)

levels(full1$Income2)

full1$Income2<-factor(full1$Income2,levels(full1$Income2)[c(2,1,3)])

levels(full1$Income2)

#recode occupation

full1$Occupation2<-NA

full1$Occupation2[full1$Occupation=="Property manager"| full1$Occupation== "Research manager"| full1$Occupation== "Dentist"| full1$Occupation== "veterinary surgeon"| full1$Occupation== "Self employed"| full1$Occupation== "GP trainee"| full1$Occupation== "Project Manager"| full1$Occupation== "Quality Systems Analyst"| full1$Occupation== "Head of Year (pastoral role in secondary education)"| full1$Occupation== "Marketing Manager"| full1$Occupation== "Data analyst"| full1$Occupation== "Civil Engineer"| full1$Occupation== "Policy Manager for NHS England"| full1$Occupation== "Critical Care Outreach Practitioner"| full1$Occupation== "Town planner"| full1$Occupation== "Doctor"| full1$Occupation== "Property manager"| full1$Occupation== "Research and Finance"| full1$Occupation== "Scientist"| full1$Occupation== "NHS Senior Improvement Advisor"| full1$Occupation== "Clinical psychologist"| full1$Occupation== "Recruitment consultant"| full1$Occupation== "Fe lecturer"| full1$Occupation== "Lecturer & researcher"| full1$Occupation== "Portfolio Research Facilitator"| full1$Occupation== "Solicitor"| full1$Occupation== "Hospital dentist"| full1$Occupation== "Self-employed"| full1$Occupation== "Project coordinator"| full1$Occupation== "Quality Assessor"| full1$Occupation== "Business analyst"| full1$Occupation== "Manager at Manufacturing Facility"| full1$Occupation== "Chartered accountant"| full1$Occupation== "Recruitment Manager"| full1$Occupation== "Quality administration"| full1$Occupation== "Business Analyst"| full1$Occupation== "Head teacher"| full1$Occupation== "Change analyst"| full1$Occupation== "Sales Co-ordinator"| full1$Occupation== "Project Manager"| full1$Occupation== "Quality specialist"| full1$Occupation== "Business Analyst"| full1$Occupation== "Pharmacist "]<- "More advantaged groups NS-SEC groups 1-4"

full1$Occupation2[full1$Occupation== "Clinical Psychologist"| full1$Occupation== "Senior Researcher (Charity sector)"| full1$Occupation== "Anaesthetist"| full1$Occupation== "Pharmacist"| full1$Occupation== "Vet and research scientist"| full1$Occupation== "Lecturer"| full1$Occupation== "Marketing Manager"| full1$Occupation== "Solicitor"| full1$Occupation== "Director tax and accounting"| full1$Occupation== "Self employed"| full1$Occupation== "Lead HR Business Partner"| full1$Occupation== "Pharmacist "| full1$Occupation== "Veterinary Surgeon"| full1$Occupation=="Project manager"| full1$Occupation== "Urban Design Officer"| full1$Occupation== "Local Government"| full1$Occupation== "Lecturer university"| full1$Occupation== "Doctor (GP)"| full1$Occupation== "Compaby Director"| full1$Occupation== "Project Manager"| full1$Occupation== "Speech and Language Therapist"| full1$Occupation== "Management Consultant"| full1$Occupation== "Hospital Doctor"| full1$Occupation== "Nurse"| full1$Occupation== "Events manager"| full1$Occupation== "Physiotherapist"| full1$Occupation== "Investment Banker"| full1$Occupation== "Teacher"| full1$Occupation== "Teacher"| full1$Occupation== "Lab technician"| full1$Occupation== "Neonatal ANP"| full1$Occupation== "Nhs Health Care Assistant"| full1$Occupation== "Teacher"| full1$Occupation== "Occupational therapy"| full1$Occupation== "Teacher"| full1$Occupation== "Business Development Coordinator for Charitable Orgs "| full1$Occupation== "Community Heart Failure Nurse"| full1$Occupation== "Nurse"| full1$Occupation== "Librarian"| full1$Occupation=="Laboratory tech"| full1$Occupation== "Veterinary Surgeon"| full1$Occupation== "Social worker "| full1$Occupation== "Behaviour Analyst"| full1$Occupation== "ABA therapist"| full1$Occupation== "Educator"| full1$Occupation== "Supply teacher"| full1$Occupation== "Community Engagement Officer"| full1$Occupation== "Physiotherapist"| full1$Occupation== "Marketing "| full1$Occupation== "Staff Trainer"| full1$Occupation== "Account Director"| full1$Occupation== "mental health nurse"| full1$Occupation== "Communications Officer"| full1$Occupation== "Teacher"| full1$Occupation== "Nurse"| full1$Occupation== "Special needs teacher"| full1$Occupation== "Physiotherapist"| full1$Occupation== "NHS Personal Assistant"]<- "More advantaged groups NS-SEC groups 1-4"

full1$Occupation2[full1$Occupation== "Registered nurse"| full1$Occupation== "NHS"| full1$Occupation== "Communications Officer "| full1$Occupation== "District Nurse"| full1$Occupation== "Account Manager"| full1$Occupation== "Staff Nurse"| full1$Occupation== "Radiotherapist"| full1$Occupation=="Account Manager"| full1$Occupation== "Education "| full1$Occupation== "Illustrator"| full1$Occupation== "student nurse"| full1$Occupation== "Mental health nurse"| full1$Occupation== "Letting agent"| full1$Occupation== "Nurse"| full1$Occupation== "Finance assistant"| full1$Occupation== "Marketer"| full1$Occupation== "Healthcare assistant"| full1$Occupation== "Care assistant"| full1$Occupation== "Teacher"| full1$Occupation== "Therapy Radiographer"| full1$Occupation== "Manager/social worker"| full1$Occupation== "Marketing officer"| full1$Occupation== "Registered Nurse"| full1$Occupation== "Technician"| full1$Occupation== "Teacher"| full1$Occupation== "Mental health nurse"| full1$Occupation== "Teacher"| full1$Occupation== "Copywriter"| full1$Occupation== "Manager"| full1$Occupation== "Nurse"| full1$Occupation== "Supervisor retail"| full1$Occupation== "Student nurse "| full1$Occupation== "Teacher"| full1$Occupation== "MIS Officer"| full1$Occupation== "Specialist Nurse"| full1$Occupation== "Care assistant"| full1$Occupation== "Customer services manager"| full1$Occupation== "Financial Advisor "| full1$Occupation== "Nurse"| full1$Occupation== "Therapy assistant"| full1$Occupation=="Nursery deputy manager"| full1$Occupation== "Nurse"| full1$Occupation== "Learning disability nurse"| full1$Occupation== "Video Producer"| full1$Occupation== "Healthcare assistant"| full1$Occupation== "Process Technologist (Food Manufacturing)"| full1$Occupation== "Nurse"| full1$Occupation== "Teacher"| full1$Occupation== "Business development manager "| full1$Occupation== "Manager"| full1$Occupation== "Training Officer"| full1$Occupation== "Teacher"| full1$Occupation== "Nurse"| full1$Occupation== "Nursery worker"| full1$Occupation== "Student Midwife"| full1$Occupation== "Housing officer"| full1$Occupation== "Nurse"| full1$Occupation== "Registered nurse"| full1$Occupation== "Business Development Coordinator"| full1$Occupation== "Student Money Adviser"| full1$Occupation== "Senior Healthcare Assistant"| full1$Occupation== "Teacher"| full1$Occupation== "Care coordinator NHS"| full1$Occupation== "Research assistant"| full1$Occupation== "Bank manager"] <- "More advantaged groups NS-SEC groups 1-4"

full1$Occupation2[full1$Occupation== "Supply teacher"| full1$Occupation== "Physiotherapist"| full1$Occupation== "SEN teacher "| full1$Occupation=="Healthcare assistant"| full1$Occupation== "Supervisor at an Investment Bank"| full1$Occupation== "Looked After Children`s Nurse"| full1$Occupation== "Police officer"| full1$Occupation== "Agency"| full1$Occupation== "Administration"| full1$Occupation== "Local goverment officer"| full1$Occupation== "Finance Administrator"| full1$Occupation== "Client account administration"| full1$Occupation== "Operations Coordinator"| full1$Occupation== "Civil servant"| full1$Occupation== "Executive Assistant"| full1$Occupation== "Bank clerk"| full1$Occupation== "Administrator"| full1$Occupation== "Administrator"| full1$Occupation== "Police Officer"| full1$Occupation== "Care assistant"| full1$Occupation== "HR admin"| full1$Occupation== "Teaching assistant "| full1$Occupation== "Early years practitioner"| full1$Occupation== "Admin role"| full1$Occupation== "Customer servic advisor"| full1$Occupation== "Training Administrator"| full1$Occupation== "Office admin"| full1$Occupation== "Nursery nurse"| full1$Occupation== "Childcare practitioner"| full1$Occupation=="Call center operator"| full1$Occupation== "Teaching Assistant"| full1$Occupation== "Civil servant"| full1$Occupation== "Office staff"| full1$Occupation== "Administrator in the NHS"| full1$Occupation== "Customer care"| full1$Occupation== "Nursery nurse"| full1$Occupation== "Call centre"| full1$Occupation== "Teaching assistant"| full1$Occupation== "Claims Handler"| full1$Occupation== "Police Staff"| full1$Occupation== "Marine administrator"| full1$Occupation== "business banker"| full1$Occupation== "Special Needs Teaching Assistant"| full1$Occupation== "Designer"| full1$Occupation== "Licensee"] <- "More advantaged groups NS-SEC groups 1-4"

full1$Occupation2[is.na(full1$Occupation2)] <- "Less advantaged groups NS-SEC groups 5-8"

full1$Occupation2[full1$Occupation=="Student" |full1$Occupation== "Dog Walker and student" |is.na(full1$Occupation) |full1$Occupation=="Postgraduate student"]<-"Exceptions"

#reorder SEC

full1$Occupation2<-as.factor(full1$Occupation2)

levels(full1$Occupation2)

full1$Occupation2<-factor(full1$Occupation2,levels(full1$Occupation2)[c(3,2,1)])

levels(full1$Occupation2)

#Create a new field for ethnicity

full1$Ethnicity2<-"BAME"

full1$Ethnicity2[full1$Ethnicity=="1"]<-"White British"

full1$Ethnicity2[full1$Ethnicity=="2" | full1$Ethnicity=="3" | full1$Ethnicity=="4"]<-"White Other"

#reorder ethnicity

full1$Ethnicity2<-as.factor(full1$Ethnicity2)

levels(full1$Ethnicity2)

full1$Ethnicity2<-factor(full1$Ethnicity2,levels(full1$Ethnicity2)[c(2,3,1)])

levels(full1$Ethnicity2)

#create new field for number of children

full1$No.of.children2<-NA

full1$No.of.children2[full1$No.of.children=="1"]<-"This is my first"

full1$No.of.children2[full1$No.of.children=="2"]<-"1"

full1$No.of.children2[full1$No.of.children=="3"]<-"2"

full1$No.of.children2[full1$No.of.children=="4" | full1$No.of.children=="5"]<-"3+"

#reorder number of children

full1$No.of.children2<-as.factor(full1$No.of.children2)

levels(full1$No.of.children2)

full1$No.of.children2<-factor(full1$No.of.children2,levels(full1$No.of.children2)[c(4,1,2,3)])

levels(full1$No.of.children2)

#create new field for number in household

full1$No.in.household2<-NA

full1$No.in.household2[full1$No.in.household=="1"]<-"1"

full1$No.in.household2[full1$No.in.household=="2"]<-"2"

full1$No.in.household2[full1$No.in.household=="3"]<-"3"

full1$No.in.household2[full1$No.in.household=="4" | full1$No.in.household=="5" | full1$No.in.household=="8"]<-"4+"

#put weeks pregnant in numeric

full1$Weeks.pregnant<-as.numeric(full1$Weeks.pregnant)

#pertussis

full1$Pertussis<-NA

full1$Pertussis[full1$Pertuss.vacc.yes=="1"]<-"Yes"

full1$Pertussis[full1$Pertuss.vacc.no=="1"]<-"No"

#reorder pertussis

full1$Pertussis<-as.factor(full1$Pertussis)

levels(full1$Pertussis)

full1$Pertussis<-factor(full1$Pertussis,levels(full1$Pertussis)[c(2,1)])

levels(full1$Pertussis)

#recode education

full1$Education2<-NA

full1$Education2[full1$Education.Lvl=="1"]<-"Below GCSE"

full1$Education2[full1$Education.Lvl=="2"]<-"GCSE or similar"

full1$Education2[full1$Education.Lvl=="3"]<-"NVQ or similar"

full1$Education2[full1$Education.Lvl=="4"]<-"A-level or similar"

full1$Education2[full1$Education.Lvl=="5"]<-"Undergraduate"

full1$Education2[full1$Education.Lvl=="6"]<-"Postgraduate"

full1$Education2[full1$Education.Lvl=="7"]<-"Other"

#reorder education

full1$Education2<-as.factor(full1$Education2)

levels(full1$Education2)

full1$Education2<-factor(full1$Education2,levels(full1$Education2)[c(2,3,1,6,5,4)])

levels(full1$Education2)

#put high risk in numeric

##full1$High.risk<-as.numeric(full1$High.risk)

#new flu vaccine field

full1$final.flu.vacc.this.Preg<-NA

full1$final.flu.vacc.this.Preg <- full1$Flu.vacc.this.preg

full1$final.flu.vacc.this.Preg[full1$Flu.vacc.this.preg=="2" & full1$If.no.intend=="1"] <- "1"

table(full1$Flu.vacc.this.preg,full1$If.no.intend)

#recode OTC medicine

full1$OTC.meds.flu2<-NA

full1$OTC.meds.flu2[full1$OTC.meds.flu=="1"]<-"Yes"

full1$OTC.meds.flu2[full1$OTC.meds.flu=="2"]<-"No"

#reorder OTC medicine

full1$OTC.meds.flu2<-as.factor(full1$OTC.meds.flu2)

levels(full1$OTC.meds.flu2)

full1$OTC.meds.flu2<-factor(full1$OTC.meds.flu2,levels(full1$OTC.meds.flu2)[c(2,1)])

levels(full1$OTC.meds.flu2)

#recode alternative flu medicine

full1$Alt.med.flu2<-NA

full1$Alt.med.flu2[full1$Alt.med.flu=="1"]<-"Yes"

full1$Alt.med.flu2[full1$Alt.med.flu=="2"]<-"No"

#reorder alternative flu medicine

full1$Alt.med.flu2<-as.factor(full1$Alt.med.flu2)

levels(full1$Alt.med.flu2)

full1$Alt.med.flu2<-factor(full1$Alt.med.flu2,levels(full1$Alt.med.flu2)[c(2,1)])

levels(full1$Alt.med.flu2)

#recode antenatal vitamins

full1$Antenatal.vitamins2<-NA

full1$Antenatal.vitamins2[full1$Antenatal.vitamins=="1"]<-"Yes"

full1$Antenatal.vitamins2[full1$Antenatal.vitamins=="2"]<-"No"

#reorder antenatal vitamins

full1$Antenatal.vitamins2<-as.factor(full1$Antenatal.vitamins2)

levels(full1$Antenatal.vitamins2)

full1$Antenatal.vitamins2<-factor(full1$Antenatal.vitamins2,levels(full1$Antenatal.vitamins2)[c(2,1)])

levels(full1$Antenatal.vitamins2)

#recode folic acid

full1$Folic.acid2<-NA

full1$Folic.acid2[full1$Folic.acid=="1"]<-"Yes"

full1$Folic.acid2[full1$Folic.acid=="2"]<-"No"

#reorder folic acid

full1$Folic.acid2<-as.factor(full1$Folic.acid2)

levels(full1$Folic.acid2)

full1$Folic.acid2<-factor(full1$Folic.acid2,levels(full1$Folic.acid2)[c(2,1)])

levels(full1$Folic.acid2)

#reorder if COVID vaccine available would you have it

full1$If.COVID.vacc.available.would.have<-as.factor(full1$If.COVID.vacc.available.would.have)

levels(full1$If.COVID.vacc.available.would.have)

full1$If.COVID.vacc.available.would.have<-factor(full1$If.COVID.vacc.available.would.have,levels(full1$If.COVID.vacc.available.would.have)[c(5,2,3,1,4)])

levels(full1$If.COVID.vacc.available.would.have)

#recode likert scales

full1<-full1 %>%

mutate_at(c(68:122), funs(recode(., `Strongly Disagree`="Disagree", `Disagree`="Disagree", `Neither Agree or Disagree`="Neither Agree or Disagree", `Agree`="Agree", `Strongly Agree`="Agree", .default = as.character(NA))))

#recode high risk

full1$High.risk2<-NA

full1$High.risk2[full1$High.risk=="1"]<-"Yes"

full1$High.risk2[full1$High.risk=="2"]<-"No"

#reorder high risk

full1$High.risk2<-as.factor(full1$High.risk2)

levels(full1$High.risk2)

full1$High.risk2<-factor(full1$High.risk2,levels(full1$High.risk2)[c(2,1)])

levels(full1$High.risk2)

###reorder likert questions###

full1$Flu.ill<-as.factor(full1$Flu.ill)

levels(full1$Flu.ill)

full1$Flu.ill<-factor(full1$Flu.ill,levels(full1$Flu.ill)[c(2,3,1)])

levels(full1$Flu.ill)

#_______________________________________________________________________________________________________

full1$Flu.stay.home.work.school<-as.factor(full1$Flu.stay)

levels(full1$Flu.stay.home.work.school)

full1$Flu.stay.home.work.school<-factor(full1$Flu.stay.home.work.school,levels(full1$Flu.stay.home.work.school)[c(2,3,1)])

levels(full1$Flu.stay.home.work.school)

#_______________________________________________________________________________________________________

full1$Flu.baby.ill<-as.factor(full1$Flu.baby.ill)

levels(full1$Flu.baby.ill)

full1$Flu.baby.ill<-factor(full1$Flu.baby.ill,levels(full1$Flu.baby.ill)[c(2,3,1)])

levels(full1$Flu.baby.ill)

#_______________________________________________________________________________________________________

full1$Flu.hurt.baby<-as.factor(full1$Flu.hurt.baby)

levels(full1$Flu.hurt.baby)

full1$Flu.hurt.baby<-factor(full1$Flu.hurt.baby,levels(full1$Flu.hurt.baby)[c(2,3,1)])

levels(full1$Flu.hurt.baby)

#_______________________________________________________________________________________________________

full1$Flu.family.friends.ill<-as.factor(full1$Flu.family.friends.ill)

levels(full1$Flu.family.friends.ill)

full1$Flu.family.friends.ill<-factor(full1$Flu.family.friends.ill,levels(full1$Flu.family.friends.ill)[c(2,3,1)])

levels(full1$Flu.family.friends.ill)

#_______________________________________________________________________________________________________

full1$Flu.coworkers.colleages.ill<-as.factor(full1$Flu.coworkers.colleages.ill)

levels(full1$Flu.coworkers.colleages.ill)

full1$Flu.coworkers.colleages.ill<-factor(full1$Flu.coworkers.colleages.ill,levels(full1$Flu.coworkers.colleages.ill)[c(2,3,1)])

levels(full1$Flu.coworkers.colleages.ill)

#_______________________________________________________________________________________________________

full1$Flu.die<-as.factor(full1$Flu.die)

levels(full1$Flu.die)

full1$Flu.die<-factor(full1$Flu.die,levels(full1$Flu.die)[c(2,3,1)])

levels(full1$Flu.die)

#_______________________________________________________________________________________________________

full1$Knowledgeable.flu.general<-as.factor(full1$Knowledgeable.flu.general)

levels(full1$Knowledgeable.flu.general)

full1$Knowledgeable.flu.general<-factor(full1$Knowledgeable.flu.general,levels(full1$Knowledgeable.flu.general)[c(2,3,1)])

levels(full1$Knowledgeable.flu.general)

#_______________________________________________________________________________________________________

full1$Knowledgeable.flu.risk<-as.factor(full1$Knowledgeable.flu.risk)

levels(full1$Knowledgeable.flu.risk)

full1$Knowledgeable.flu.risk<-factor(full1$Knowledgeable.flu.risk,levels(full1$Knowledgeable.flu.risk)[c(2,3,1)])

levels(full1$Knowledgeable.flu.risk)

#_______________________________________________________________________________________________________

full1$I.am.risk.getting.flu<-as.factor(full1$I.am.risk.getting.flu)

levels(full1$I.am.risk.getting.flu)

full1$I.am.risk.getting.flu<-factor(full1$I.am.risk.getting.flu,levels(full1$I.am.risk.getting.flu)[c(2,3,1)])

levels(full1$I.am.risk.getting.flu)

#_______________________________________________________________________________________________________

full1$Family.friends.risk.getting.flu<-as.factor(full1$Family.friends.risk.getting.flu)

levels(full1$Family.friends.risk.getting.flu)

full1$Family.friends.risk.getting.flu<-factor(full1$Family.friends.risk.getting.flu,levels(full1$Family.friends.risk.getting.flu)[c(2,3,1)])

levels(full1$Family.friends.risk.getting.flu)

#_______________________________________________________________________________________________________

full1$Flu.vacc.side.effects<-as.factor(full1$Flu.vacc.side.effects)

levels(full1$Flu.vacc.side.effects)

full1$Flu.vacc.side.effects<-factor(full1$Flu.vacc.side.effects,levels(full1$Flu.vacc.side.effects)[c(2,3,1)])

levels(full1$Flu.vacc.side.effects)

#_______________________________________________________________________________________________________

full1$Flu.vacc.ill<-as.factor(full1$Flu.vacc.ill)

levels(full1$Flu.vacc.ill)

full1$Flu.vacc.ill<-factor(full1$Flu.vacc.ill,levels(full1$Flu.vacc.ill)[c(2,3,1)])

levels(full1$Flu.vacc.ill)

#_______________________________________________________________________________________________________

full1$Flu.vacc.hurt.baby<-as.factor(full1$Flu.vacc.hurt.baby)

levels(full1$Flu.vacc.hurt.baby)

full1$Flu.vacc.hurt.baby<-factor(full1$Flu.vacc.hurt.baby,levels(full1$Flu.vacc.hurt.baby)[c(2,3,1)])

levels(full1$Flu.vacc.hurt.baby)

#_______________________________________________________________________________________________________

full1$Flu.vacc.painful<-as.factor(full1$Flu.vacc.painful)

levels(full1$Flu.vacc.painful)

full1$Flu.vacc.painful<-factor(full1$Flu.vacc.painful,levels(full1$Flu.vacc.painful)[c(2,3,1)])

levels(full1$Flu.vacc.painful)

#_______________________________________________________________________________________________________

full1$Flu.vacc.not.protect.me<-as.factor(full1$Flu.vacc.not.protect.me)

levels(full1$Flu.vacc.not.protect.me)

full1$Flu.vacc.not.protect.me<-factor(full1$Flu.vacc.not.protect.me,levels(full1$Flu.vacc.not.protect.me)[c(2,3,1)])

levels(full1$Flu.vacc.not.protect.me)

#_______________________________________________________________________________________________________

full1$Flu.vacc.not.protect.baby<-as.factor(full1$Flu.vacc.not.protect.baby)

levels(full1$Flu.vacc.not.protect.baby)

full1$Flu.vacc.not.protect.baby<-factor(full1$Flu.vacc.not.protect.baby,levels(full1$Flu.vacc.not.protect.baby)[c(2,3,1)])

levels(full1$Flu.vacc.not.protect.baby)

#_______________________________________________________________________________________________________

full1$Inconvenient.flu.vacc<-as.factor(full1$Inconvenient.flu.vacc)

levels(full1$Inconvenient.flu.vacc)

full1$Inconvenient.flu.vacc<-factor(full1$Inconvenient.flu.vacc,levels(full1$Inconvenient.flu.vacc)[c(2,3,1)])

levels(full1$Inconvenient.flu.vacc)

#_______________________________________________________________________________________________________

full1$Shortage.flu.vacc<-as.factor(full1$Shortage.flu.vacc)

levels(full1$Shortage.flu.vacc)

full1$Shortage.flu.vacc<-factor(full1$Shortage.flu.vacc,levels(full1$Shortage.flu.vacc)[c(2,3,1)])

levels(full1$Shortage.flu.vacc)

#_______________________________________________________________________________________________________

full1$Flu.vacc.recommended<-as.factor(full1$Flu.vacc.recommended)

levels(full1$Flu.vacc.recommended)

full1$Flu.vacc.recommended<-factor(full1$Flu.vacc.recommended,levels(full1$Flu.vacc.recommended)[c(2,3,1)])

levels(full1$Flu.vacc.recommended)

#_______________________________________________________________________________________________________

full1$Flu.vacc.not.get.ill<-as.factor(full1$Flu.vacc.not.get.ill)

levels(full1$Flu.vacc.not.get.ill)

full1$Flu.vacc.not.get.ill<-factor(full1$Flu.vacc.not.get.ill,levels(full1$Flu.vacc.not.get.ill)[c(2,3,1)])

levels(full1$Flu.vacc.not.get.ill)

#_______________________________________________________________________________________________________

full1$Flu.vacc.prevent.baby.ill<-as.factor(full1$Flu.vacc.prevent.baby.ill)

levels(full1$Flu.vacc.prevent.baby.ill)

full1$Flu.vacc.prevent.baby.ill<-factor(full1$Flu.vacc.prevent.baby.ill,levels(full1$Flu.vacc.prevent.baby.ill)[c(2,3,1)])

levels(full1$Flu.vacc.prevent.baby.ill)

#_______________________________________________________________________________________________________

full1$Flu.vacc.prevent.family.friends.ill<-as.factor(full1$Flu.vacc.prevent.family.friends.ill)

levels(full1$Flu.vacc.prevent.family.friends.ill)

full1$Flu.vacc.prevent.family.friends.ill<-factor(full1$Flu.vacc.prevent.family.friends.ill,levels(full1$Flu.vacc.prevent.family.friends.ill)[c(2,3,1)])

levels(full1$Flu.vacc.prevent.family.friends.ill)

#_______________________________________________________________________________________________________

full1$Vacc.prevent.disease<-as.factor(full1$Vacc.prevent.disease)

levels(full1$Vacc.prevent.disease)

full1$Vacc.prevent.disease<-factor(full1$Vacc.prevent.disease,levels(full1$Vacc.prevent.disease)[c(2,3,1)])

levels(full1$Vacc.prevent.disease)

#_______________________________________________________________________________________________________

full1$Vacc.safe<-as.factor(full1$Vacc.safe)

levels(full1$Vacc.safe)

full1$Vacc.safe<-factor(full1$Vacc.safe,levels(full1$Vacc.safe)[c(2,3,1)])

levels(full1$Vacc.safe)

#_______________________________________________________________________________________________________

full1$Intend.vacc.child.flu<-as.factor(full1$Intend.vacc.child.flu)

levels(full1$Intend.vacc.child.flu)

full1$Intend.vacc.child.flu<-factor(full1$Intend.vacc.child.flu,levels(full1$Intend.vacc.child.flu)[c(2,3,1)])

levels(full1$Intend.vacc.child.flu)

#_______________________________________________________________________________________________________

full1$Intend.vacc.baby<-as.factor(full1$Intend.vacc.baby)

levels(full1$Intend.vacc.baby)

full1$Intend.vacc.baby<-factor(full1$Intend.vacc.baby,levels(full1$Intend.vacc.baby)[c(2,3,1)])

levels(full1$Intend.vacc.baby)

#_______________________________________________________________________________________________________

full1$More.likely.vacc.if.family.friends.have<-as.factor(full1$More.likely.vacc.if.family.friends.have)

levels(full1$More.likely.vacc.if.family.friends.have)

full1$More.likely.vacc.if.family.friends.have<-factor(full1$More.likely.vacc.if.family.friends.have,levels(full1$More.likely.vacc.if.family.friends.have)[c(2,3,1)])

levels(full1$More.likely.vacc.if.family.friends.have)

#_______________________________________________________________________________________________________

full1$Doc.flu<-as.factor(full1$Doc.flu)

levels(full1$Doc.flu)

full1$Doc.flu<-factor(full1$Doc.flu,levels(full1$Doc.flu)[c(2,3,1)])

levels(full1$Doc.flu)

#_______________________________________________________________________________________________________

full1$Pharm.flu<-as.factor(full1$Pharm.flu)

levels(full1$Pharm.flu)

full1$Pharm.flu<-factor(full1$Pharm.flu,levels(full1$Pharm.flu)[c(2,3,1)])

levels(full1$Pharm.flu)

#_______________________________________________________________________________________________________

full1$Nurse.flu<-as.factor(full1$Nurse.flu)

levels(full1$Nurse.flu)

full1$Nurse.flu<-factor(full1$Nurse.flu,levels(full1$Nurse.flu)[c(2,3,1)])

levels(full1$Nurse.flu)

#_______________________________________________________________________________________________________

full1$Midwife.flu<-as.factor(full1$Midwife.flu)

levels(full1$Midwife.flu)

full1$Midwife.flu<-factor(full1$Midwife.flu,levels(full1$Midwife.flu)[c(2,3,1)])

levels(full1$Midwife.flu)

#_______________________________________________________________________________________________________

full1$Health.visitor.flu<-as.factor(full1$Health.visitor.flu)

levels(full1$Health.visitor.flu)

full1$Health.visitor.flu<-factor(full1$Health.visitor.flu,levels(full1$Health.visitor.flu)[c(2,3,1)])

levels(full1$Health.visitor.flu)

#_______________________________________________________________________________________________________

full1$Fam.friend.flu<-as.factor(full1$Fam.friend.flu)

levels(full1$Fam.friend.flu)

full1$Fam.friend.flu<-factor(full1$Fam.friend.flu,levels(full1$Fam.friend.flu)[c(2,3,1)])

levels(full1$Fam.friend.flu)

#_______________________________________________________________________________________________________

full1$COVID.ill<-as.factor(full1$COVID.ill)

levels(full1$COVID.ill)

full1$COVID.ill<-factor(full1$COVID.ill,levels(full1$COVID.ill)[c(2,3,1)])

levels(full1$COVID.ill)

#_______________________________________________________________________________________________________

full1$COVID.isolate<-as.factor(full1$COVID.isolate)

levels(full1$COVID.isolate)

full1$COVID.isolate<-factor(full1$COVID.isolate,levels(full1$COVID.isolate)[c(2,3,1)])

levels(full1$COVID.isolate)

#_______________________________________________________________________________________________________

full1$COVID.friends<-as.factor(full1$COVID.friends)

levels(full1$COVID.friends)

full1$COVID.friends<-factor(full1$COVID.friends,levels(full1$COVID.friends)[c(2,3,1)])

levels(full1$COVID.friends)

#_______________________________________________________________________________________________________

full1$COVID.could.contact.quarantine<-as.factor(full1$COVID.could.contact.quarantine)

levels(full1$COVID.could.contact.quarantine)

full1$COVID.could.contact.quarantine<-factor(full1$COVID.could.contact.quarantine,levels(full1$COVID.could.contact.quarantine)[c(2,3,1)])

levels(full1$COVID.could.contact.quarantine)

#_______________________________________________________________________________________________________

full1$COVID.family.friends.contact.quarantine<-as.factor(full1$COVID.family.friends.contact.quarantine)

levels(full1$COVID.family.friends.contact.quarantine)

full1$COVID.family.friends.contact.quarantine<-factor(full1$COVID.family.friends.contact.quarantine,levels(full1$COVID.family.friends.contact.quarantine)[c(2,3,1)])

levels(full1$COVID.family.friends.contact.quarantine)

#_______________________________________________________________________________________________________

full1$Knowledge.COVID.general<-as.factor(full1$Knowledge.COVID.general)

levels(full1$Knowledge.COVID.general)

full1$Knowledge.COVID.general<-factor(full1$Knowledge.COVID.general,levels(full1$Knowledge.COVID.general)[c(2,3,1)])

levels(full1$Knowledge.COVID.general)

#_______________________________________________________________________________________________________

full1$Knowledge.COVID.risk<-as.factor(full1$Knowledge.COVID.risk)

levels(full1$Knowledge.COVID.risk)

full1$Knowledge.COVID.risk<-factor(full1$Knowledge.COVID.risk,levels(full1$Knowledge.COVID.risk)[c(2,3,1)])

levels(full1$Knowledge.COVID.risk)

#_______________________________________________________________________________________________________

full1$I.am.risk.getting.COVID<-as.factor(full1$I.am.risk.getting.COVID)

levels(full1$I.am.risk.getting.COVID)

full1$I.am.risk.getting.COVID<-factor(full1$I.am.risk.getting.COVID,levels(full1$I.am.risk.getting.COVID)[c(2,3,1)])

levels(full1$I.am.risk.getting.COVID)

#_______________________________________________________________________________________________________

full1$Family.friends.risk.getting.COVID<-as.factor(full1$Family.friends.risk.getting.COVID)

levels(full1$Family.friends.risk.getting.COVID)

full1$Family.friends.risk.getting.COVID<-factor(full1$Family.friends.risk.getting.COVID,levels(full1$Family.friends.risk.getting.COVID)[c(2,3,1)])

levels(full1$Family.friends.risk.getting.COVID)

#_______________________________________________________________________________________________________

full1$COVID.vacc.protect.me<-as.factor(full1$COVID.vacc.protect.me)

levels(full1$COVID.vacc.protect.me)

full1$COVID.vacc.protect.me<-factor(full1$COVID.vacc.protect.me,levels(full1$COVID.vacc.protect.me)[c(2,3,1)])

levels(full1$COVID.vacc.protect.me)

#_______________________________________________________________________________________________________

full1$COVID.vacc.protect.baby.fam.friends<-as.factor(full1$COVID.vacc.protect.baby.fam.friends)

levels(full1$COVID.vacc.protect.baby.fam.friends)

full1$COVID.vacc.protect.baby.fam.friends<-factor(full1$COVID.vacc.protect.baby.fam.friends,levels(full1$COVID.vacc.protect.baby.fam.friends)[c(2,3,1)])

levels(full1$COVID.vacc.protect.baby.fam.friends)

#_______________________________________________________________________________________________________

full1$Would.vacc.baby.COVID<-as.factor(full1$Would.vacc.baby.COVID)

levels(full1$Would.vacc.baby.COVID)

full1$Would.vacc.baby.COVID<-factor(full1$Would.vacc.baby.COVID,levels(full1$Would.vacc.baby.COVID)[c(2,3,1)])

levels(full1$Would.vacc.baby.COVID)

#_______________________________________________________________________________________________________

full1$If.COVID.vacc.seasonal.would.have<-as.factor(full1$If.COVID.vacc.seasonal.would.have)

levels(full1$If.COVID.vacc.seasonal.would.have)

full1$If.COVID.vacc.seasonal.would.have<-factor(full1$If.COVID.vacc.seasonal.would.have,levels(full1$If.COVID.vacc.seasonal.would.have)[c(2,3,1)])

levels(full1$If.COVID.vacc.seasonal.would.have)

#_______________________________________________________________________________________________________

full1$Would.have.COVID.vacc.if.not.preg<-as.factor(full1$Would.have.COVID.vacc.if.not.preg)

levels(full1$Would.have.COVID.vacc.if.not.preg)

full1$Would.have.COVID.vacc.if.not.preg<-factor(full1$Would.have.COVID.vacc.if.not.preg,levels(full1$Would.have.COVID.vacc.if.not.preg)[c(2,3,1)])

levels(full1$Would.have.COVID.vacc.if.not.preg)

#_______________________________________________________________________________________________________

full1$Doc.COVID<-as.factor(full1$Doc.COVID)

levels(full1$Doc.COVID)

full1$Doc.COVID<-factor(full1$Doc.COVID,levels(full1$Doc.COVID)[c(2,3,1)])

levels(full1$Doc.COVID)

#_______________________________________________________________________________________________________

full1$Pharm.COVID<-as.factor(full1$Pharm.COVID)

levels(full1$Pharm.COVID)

full1$Pharm.COVID<-factor(full1$Pharm.COVID,levels(full1$Pharm.COVID)[c(2,3,1)])

levels(full1$Pharm.COVID)

#_______________________________________________________________________________________________________

full1$Nurse.COVID<-as.factor(full1$Nurse.COVID)

levels(full1$Nurse.COVID)

full1$Nurse.COVID<-factor(full1$Nurse.COVID,levels(full1$Nurse.COVID)[c(2,3,1)])

levels(full1$Nurse.COVID)

#_______________________________________________________________________________________________________

full1$Midwife.COVID<-as.factor(full1$Midwife.COVID)

levels(full1$Midwife.COVID)

full1$Midwife.COVID<-factor(full1$Midwife.COVID,levels(full1$Midwife.COVID)[c(2,3,1)])

levels(full1$Midwife.COVID)

#_______________________________________________________________________________________________________

full1$Health.visitor.COVID<-as.factor(full1$Health.visitor.COVID)

levels(full1$Health.visitor.COVID)

full1$Health.visitor.COVID<-factor(full1$Health.visitor.COVID,levels(full1$Health.visitor.COVID)[c(2,3,1)])

levels(full1$Health.visitor.COVID)

#_______________________________________________________________________________________________________

full1$Fam.friend.COVID<-as.factor(full1$Fam.friend.COVID)

levels(full1$Fam.friend.COVID)

full1$Fam.friend.COVID<-factor(full1$Fam.friend.COVID,levels(full1$Fam.friend.COVID)[c(2,3,1)])

levels(full1$Fam.friend.COVID)

#recode flu vacc to include those who intend

full1$fluyes<-NA

full1$fluyes[full1$Flu.vacc.this.preg==1]<-"1"

full1$fluyes[full1$Flu.vacc.this.preg==2]<-"2"

full1$fluyes[full1$If.no.intend==1]<-"1"

full1$fluyes[full1$If.no.intend==2]<-"2"

full1$fluyes[full1$Flu.vacc.this.preg==2 & is.na(full1$If.no.intend)]<-NA

#reorder month response

levels(full1$monthresponse)

full1$monthresponse<-factor(full1$monthresponse,levels(full1$monthresponse)[c(6,2,4,3,5,1)])

levels(full1$monthresponse)

#post April 16 as factor

full1$PostApril16 <- as.factor(full1$PostApril16)

#######################################descriptive tables##############################################

#_______

#table1(~ Access.GP.online.appts + Access.GP.online.prescrp + Access.GP.online.records + Access.GP.online.other + Live.with.alone + Live.with.partner + Live.with.child.ren + Live.with.parent.s + Live.with.other.family + Live.with.roommate.s, data=full1)

#table1(~ Heard.Uni.site + Heard.GP.prac + Heard.Mumsnet + Heard.Netmums + Heard.Bambis + Heard.Liv.Mums + Heard.fam.friend + Heard.other + Heard.ques.Other.specify, data=full1)

table1(~ Flu.vacc.this.preg + If.no.intend + Flu.vacc.prev.preg + If.yes.side.effects, data=full1)

#Don`t use this

#require(rmarkdown)

#table_one <- tableby(formula = as.factor(Flu.vacc.this.preg) ~ Age+Income2+fe(Ethnicity2), data = full1,control = tableby.control(numeric.stats="medianq1q3"))

#summary(table_one)

#write2html(table_one, "~/trash.html")

#write2word(table_one, "~/trash.doc", title="My table in Word")

table1(~ If.COVID.vacc.available.would.have | You.or.close.friend.fam.pos.COVID, data=full1)

table1(~ If.COVID.vacc.available.would.have | You.or.close.friend.fam.hosp.COVID, data=full1)

table1(~ fluyes | You.or.close.friend.fam.flu, data=full1)

tab1<-tableby(fluyes ~ Age + Weeks.pregnant, data=full1, control=tableby.control( numeric.stats=c("N", "median", "q1q3")))

###################################### descriptive plots ##############################################

hist(full1$Age, main = "", xlab = "Age (weeks)")

mean(full1$Age)

min(full1$Age)

max(full1$Age)

hist(full1$Weeks.pregnant, main = "", xlab = "Weeks Pregnant")

hist(full1$High.risk)

summary(full1$Age)

IQR(full1$Age)

summary(full1$Weeks.pregnant)

IQR(full1$Weeks.pregnant)

fisher.test

#################################tables and stats####################################################################################

#install.packages("tableone")

require(tableone)

#table and stats for table 1 demographics in relation to flu vax status and willingness to receive COVID vaccine

#demographic table

table1(~ Age + Weeks.pregnant + Occupation2 +Income+ Income2+Ethnicity+ Ethnicity2+No.of.children2+No.of.children+No.in.household+No.in.household2+OTC.meds.flu2+Alt.med.flu2+Antenatal.vitamins2+Folic.acid2+Pertussis+Education.Lvl+Education2 +If.COVID.vacc.available.would.have +monthresponse + PostApril16, data=full1)

#demographic table in relation to willingness to receive COVID vax

table1(~ Age + Weeks.pregnant + Occupation2 +Income+ Income2+ Ethnicity2+No.of.children2+No.in.household2+OTC.meds.flu2+Alt.med.flu2+Antenatal.vitamins2+Folic.acid2+Pertussis+Education2+If.no.intend+Flu.vacc.this.preg +monthresponse + PostApril16| If.COVID.vacc.available.would.have, data=full1)

#summary of demographics in relation to flu vax status

summary(Flu.vacc.this.preg ~ Age + Weeks.pregnant + Occupation2 + Income2 + Education2 + Ethnicity2 + No.of.children2 + No.in.household2 + Pertussis + Antenatal.vitamins2 + High.risk2 + OTC.meds.flu2 + Alt.med.flu2 + monthresponse + PostApril16, data=full1, method="reverse", test=T)

#summary of demographics in relation to willingness to received covid vax

summary( If.COVID.vacc.available.would.have ~ Age + Weeks.pregnant + Occupation2 +Income+ Income2+ Ethnicity2+No.of.children2+No.in.household2+OTC.meds.flu+Alt.med.flu+Antenatal.vitamins+Folic.acid+Pertussis+Education2+If.no.intend+Flu.vacc.this.preg + monthresponse + PostApril16, data=full1, method="reverse", test=T)

summary(If.COVID.vacc.available.would.have ~ Age + Weeks.pregnant + Occupation2 + Income2+ Ethnicity2+No.of.children2+No.in.household2+OTC.meds.flu2+Alt.med.flu2+Antenatal.vitamins+Pertussis+Education2+If.no.intend+Flu.vacc.this.preg + High.risk2 + monthresponse + PostApril16, data=full1, method="reverse", test=T)

#stats for create table one for demographics in relation to willingness to receive covid vax

myvarstable1 <- c("Age", "Weeks.pregnant", "Occupation2", "Income2", "Ethnicity2", "No.of.children2", "No.in.household2", "OTC.meds.flu2", "Alt.med.flu2", "Antenatal.vitamins", "Folic.acid2", "Pertussis", "Education2", "If.no.intend", "Flu.vacc.this.preg", "High.risk2", "Smartphone", "monthresponse", "PostApril16")

catvarstable1 <- c("Occupation2", "Income2", "Ethnicity2", "No.of.children2", "No.in.household2", "OTC.meds.flu2", "Alt.med.flu2", "Antenatal.vitamins", "Folic.acid2", "Pertussis", "Education2", "If.no.intend", "Flu.vacc.this.preg", "High.risk2", "Smartphone", "monthresponse", "PostApril16")

exactvarstable1 <- c("", "Occupation2", "Ethnicity2", "No.of.children2", "No.in.household2", "Education2", "If.no.intend", "monthresponse", "PostApril16")

table1man1 <- CreateTableOne(vars = myvarstable1, data = full1, strata = "fluyes")

print(table1man1, exact = exactvarstable1, quote = TRUE)

table1man2 <- CreateTableOne(vars = myvarstable1, data = full1, strata = "If.COVID.vacc.available.would.have")

print(table1man2, exact = exactvarstable1, quote = TRUE)

#demographic table in relation to willingness to receive covid vax

table1(~ Age + Weeks.pregnant + Occupation2 + Income2 + Education2 + Ethnicity2 + No.of.children2 + No.in.household2 + Pertussis + Antenatal.vitamins2 + Folic.acid2 + High.risk2 + Shield + Smartphone + Exercise.before + Exercise.currently + Smoker + Discuss.vacc.with.partner.fam + monthresponse + as.factor(PostApril16)| If.COVID.vacc.available.would.have, data=full1)

#table and stats for table 2 attitudes and beliefs about flu perception and risks in relation to flu vax status

#summary for attitudes and beliefs about flu perception and risks in relation to flu vax status

summary(fluyes ~ Flu.ill + Flu.stay.home.work.school + Flu.baby.ill + Flu.hurt.baby+ Flu.family.friends.ill + Flu.coworkers.colleages.ill + Flu.die + Knowledgeable.flu.general + Knowledgeable.flu.risk + I.am.risk.getting.flu + Family.friends.risk.getting.flu + You.or.close.friend.fam.flu + OTC.meds.flu2 + Alt.med.flu2, data=full1, method="reverse", test=T)

#stats for create table one for attitudes and beliefs about flu perception and risks in relation to flu vax status (checking fisher exact)

myvarstable2<-c("Flu.ill", "Flu.stay.home.work.school", "Flu.baby.ill", "Flu.hurt.baby", "Flu.family.friends.ill", "Flu.coworkers.colleages.ill", "Flu.die", "Knowledgeable.flu.general", "Knowledgeable.flu.risk", "I.am.risk.getting.flu", "Family.friends.risk.getting.flu", "You.or.close.friend.fam.flu", "OTC.meds.flu2", "Alt.med.flu2")

catvarstable2<-c("Flu.ill", "Flu.stay.home.work.school", "Flu.baby.ill", "Flu.hurt.baby", "Flu.family.friends.ill", "Flu.coworkers.colleages.ill", "Flu.die", "Knowsledgeable.flu.general", "Knowledgeable.flu.risk", "I.am.risk.getting.flu", "Family.friends.risk.getting.flu", "You.or.close.friend.fam.flu", "OTC.meds.flu2", "Alt.med.flu2")

exactvarstable2<-c("Flu.stay.home.work.school", "Flu.family.friends.ill", "Flu.coworkers.colleages.ill", "Flu.die", "Knowledgeable.flu.general", "Knowledgeable.flu.risk", "Alt.med.flu2")

table2man<-CreateTableOne(vars=myvarstable2, data=full1, strata="fluyes")

print(table2man,exact = exactvarstable2, quote = TRUE)

#table and stats for table 3 attitudes and beliefs about flu vaccine in relation to flu vax status

#summary for attitudes and beliefs about flu vaccine in relation to flu vax status

summary(fluyes ~ Flu.vacc.side.effects + Flu.vacc.ill + Flu.vacc.hurt.baby + Flu.vacc.painful + Flu.vacc.not.protect.me + Flu.vacc.not.protect.baby + Inconvenient.flu.vacc + Shortage.flu.vacc + Flu.vacc.recommended + Flu.vacc.not.get.ill + Flu.vacc.prevent.baby.ill + Flu.vacc.prevent.family.friends.ill +Flu.vacc.prev.preg + If.yes.side.effects, data=full1, method="reverse", test=T)

#stats for create table one for attitudes and beliefs about flu vaccine in relation to flu vax status (checking fisher exact)

myvarstable3 <- c("Flu.vacc.side.effects", "Flu.vacc.ill", "Flu.vacc.hurt.baby", "Flu.vacc.painful", "Flu.vacc.not.protect.me", "Flu.vacc.not.protect.baby", "Inconvenient.flu.vacc", "Shortage.flu.vacc", "Flu.vacc.recommended", "Flu.vacc.not.get.ill", "Flu.vacc.prevent.baby.ill", "Flu.vacc.prevent.family.friends.ill", "Flu.vacc.prev.preg", "If.yes.side.effects")

catvarstable3 <- c("Flu.vacc.side.effects", "Flu.vacc.ill", "Flu.vacc.hurt.baby", "Flu.vacc.painful", "Flu.vacc.not.protect.me", "Flu.vacc.not.protect.baby", "Inconvenient.flu.vacc", "Shortage.flu.vacc", "Flu.vacc.recommended", "Flu.vacc.not.get.ill", "Flu.vacc.prevent.baby.ill", "Flu.vacc.prevent.family.friends.ill", "Flu.vacc.prev.preg", "If.yes.side.effects")

exactvarstable3 <- c("Flu.vacc.hurt.baby", "Inconvenient.flu.vacc", "Shortage.flu.vacc", "If.yes.side.effects")

table3man <- CreateTableOne(vars = myvarstable3, data = full1, strata = "fluyes")

print(table3man, exact = exactvarstable3, quote = TRUE)

#table and stats for table 4 attitudes and beliefs about covid and risks in relation to willingness to receive covid vax

#table for attitudes and beliefs about covid and risks in relation to willingness to receive covid vax

table1(~ COVID.ill + COVID.isolate + COVID.friends + COVID.could.contact.quarantine + COVID.family.friends.contact.quarantine + Knowledge.COVID.general + Knowledge.COVID.general + Knowledge.COVID.risk + I.am.risk.getting.COVID + Family.friends.risk.getting.COVID + You.or.close.friend.fam.pos.COVID + You.or.close.friend.fam.hosp.COVID | If.COVID.vacc.available.would.have, data=full1)

#summary for attitudes and beliefs about covid and risks in relation to willingness to receive covid vax

summary(If.COVID.vacc.available.would.have ~ COVID.ill + COVID.isolate + COVID.friends + COVID.could.contact.quarantine + COVID.family.friends.contact.quarantine + Knowledge.COVID.general + Knowledge.COVID.general + Knowledge.COVID.risk + I.am.risk.getting.COVID + Family.friends.risk.getting.COVID + You.or.close.friend.fam.pos.COVID + You.or.close.friend.fam.hosp.COVID, data=full1, method="reverse", test=T)

#stats for create table one for attitudes and beliefs about covid and risks in relation to willingness to receive covid vax (checking fisher exact)

myvarstable4 <- c("COVID.ill", "COVID.isolate", "COVID.friends", "COVID.could.contact.quarantine", "COVID.family.friends.contact.quarantine", "Knowledge.COVID.general", "Knowledge.COVID.risk", "I.am.risk.getting.COVID", "Family.friends.risk.getting.COVID", "You.or.close.friend.fam.pos.COVID", "You.or.close.friend.fam.hosp.COVID")

catvarstable4 <- c("COVID.ill", "COVID.isolate", "COVID.friends", "COVID.could.contact.quarantine", "COVID.family.friends.contact.quarantine", "Knowledge.COVID.general", "Knowledge.COVID.risk", "I.am.risk.getting.COVID", "Family.friends.risk.getting.COVID", "You.or.close.friend.fam.pos.COVID", "You.or.close.friend.fam.hosp.COVID")

exactvarstable4 <- c("COVID.ill", "COVID.isolate", "COVID.could.contact.quaratine", "COVID.family.friends.contact.quarantine", "Knowledgeable.COVID.general", "Knowledge.COVID.risk", "I.am.risk.getting.COVID", "Family.friends.risk.getting.COVID")

table4man <- CreateTableOne(vars = myvarstable4, data = full1, strata = "If.COVID.vacc.available.would.have")

print(table4man, exact = exactvarstable4, quote = TRUE)

#table for table 5 attitudes and beleiefs towards covid vax

table1(~ If.COVID.vacc.available.would.have + COVID.vacc.protect.me + COVID.vacc.protect.baby.fam.friends + Would.vacc.baby.COVID + If.COVID.vacc.seasonal.would.have + Would.have.COVID.vacc.if.not.preg, data=full1)

#table and stats for supplementary table 1 health behaviours in relation to flu vax status and willingness to receive covid vax

#stats for create table one for health behaviours in relation to flu vax status and willigness to receive covid vax (checking fisher exact); this is where pertussis info is

myvarstableS1 <- c("Pertussis", "Antenatal.vitamins2", "Folic.acid2", "High.risk2", "Shield", "Exercise.before", "Exercise.currently", "Smoker", "Discuss.vacc.with.partner.fam")

catvarstableS1 <- c("Pertussis", "Antenatal.vitamins2", "Folic.acid2", "High.risk2", "Shield", "Exercise.before", "Exercise.currently", "Smoker", "Discuss.vacc.with.partner.fam")

exactvarstableS1 <- c("Antenatal.vitamins2", "High.risk2", "Exercise.before", "Exercise.currently", "Smoker")

tableS1man1 <- CreateTableOne(vars = myvarstableS1, data = full1, strata = "fluyes")

print(tableS1man1, exact = exactvarstableS1, quote = TRUE)

tableS1man2 <- CreateTableOne(vars = myvarstableS1, data = full1, strata = "If.COVID.vacc.available.would.have")

print(tableS1man2, exact = exactvarstableS1, quote = TRUE)

#table and stats for supplementary table 2 means by which flu vax was offered

table1(~ Offered.flu.vacc + If.yes.offered.GP + If.yes.offered.wo.hosp + If.yes.offered.comm.ser.midwife + If.yes.offered.health.vis + If.yes.offered.pharm + If.yes.offered.other + Who.other.offered.specify + How.offered.letter + How.offered.text + How.offered.face.to.face + How.offered.other + How.other.offered.specify, data=full1)

#table and stats for supplementary table 3 attitudes and beliefs towards vaccination in general in relation to flu vax status and willingness to accept covid vax

#table for attitudes and beliefs towards vaccination in general in relation to flu vax status and willingness to receive covid vax

table1(~ Vacc.prevent.disease + Vacc.safe + Intend.vacc.child.flu + Intend.vacc.baby + More.likely.vacc.if.family.friends.have | If.COVID.vacc.available.would.have, data=full1)

#stats for create table one for attitudes and beliefs towards vaccination in general in relation to flu vax status and willingness to receive covid vax (Checking fisher exact)

myvarstableS3 <- c("Vacc.prevent.disease", "Vacc.safe", "Intend.vacc.child.flu", "Intend.vacc.baby", "More.likely.vacc.if.family.friends.have")

catvarstableS3 <- c("Vacc.prevent.disease", "Vacc.safe", "Intend.vacc.child.flu", "Intend.vacc.baby", "More.likely.vacc.if.family.friends.have")

exactvarstableS3 <- c("Vacc.prevent.disease", "Vacc.safe", "Intend.vacc.child.flu", "Intend.vacc.baby")

tableS3man1 <- CreateTableOne(vars = myvarstableS3, data = full1, strata = "fluyes")

print(tableS3man1, exact = exactvarstableS3, quote = TRUE)

tableS3man2 <- CreateTableOne(vars = myvarstableS3, data = full1, strata = "If.COVID.vacc.available.would.have")

print(tableS3man2, exact = exactvarstableS3, quote = TRUE)

#table and stats for supplementary table 4 likelihood to accept flu vax from different healthcare professionals in relation to flu vax status

myvarstableS4 <- c("Doc.flu", "Pharm.flu", "Nurse.flu", "Midwife.flu", "Health.visitor.flu", "Fam.friend.flu")

catvarstableS4 <- c("Doc.flu", "Pharm.flu", "Nurse.flu", "Midwife.flu", "Health.visitor.flu", "Fam.friend.flu")

exactvarstableS4 <- c("Doc.flu", "Nurse.flu", "Midwife.flu")

tableS4man <- CreateTableOne(vars = myvarstableS4, data = full1, strata = "fluyes")

print(tableS4man, exact = exactvarstableS4, quote = TRUE)

#table and stats for supplementary table 5 likelihood to accept covid vax from different healthcare professionals in relation to willingness to receive covid vax

#table for likelihood to accept covid vax from different healthcare professionals in relation to willingness to receive covid vax

table1(~ Doc.COVID + Pharm.COVID + Nurse.COVID + Midwife.COVID + Health.visitor.COVID + Fam.friend.COVID | If.COVID.vacc.available.would.have, data=full1)

#stats for create table one for likelihood to accept covid vax from different healthcare professionals in relation to willingness to receive covid vac (Checking fisher exact)

myvarstableS5 <- c("Doc.COVID", "Pharm.COVID", "Nurse.COVID", "Midwife.COVID", "Health.visitor.COVID", "Fam.friend.COVID")

catvarstableS5 <- c("Doc.COVID", "Pharm.COVID", "Nurse.COVID", "Midwife.COVID", "Health.visitor.COVID", "Fam.friend.COVID")

exactvarstableS5 <- c("Doc.COVID", "Pharm.COVID", "Nurse.COVID", "Midwife.COVID", "Health.visitor.COVID")

tableS5man <- CreateTableOne(vars = myvarstableS5, data = full1, strata = "If.COVID.vacc.available.would.have")

print(tableS5man, exact = exactvarstableS5, quote = TRUE)
